# Supplementary figures and images for: An artificial-vision- and statistical-learning-based method for studying the biodegradation of type I collagen scaffolds in bone regeneration systems
Source: PeerJ. 2019 Jul 5;7:e7233. doi: 10.7717/peerj.7233 (PMC6613533; doi:10.7717/peerj.7233)

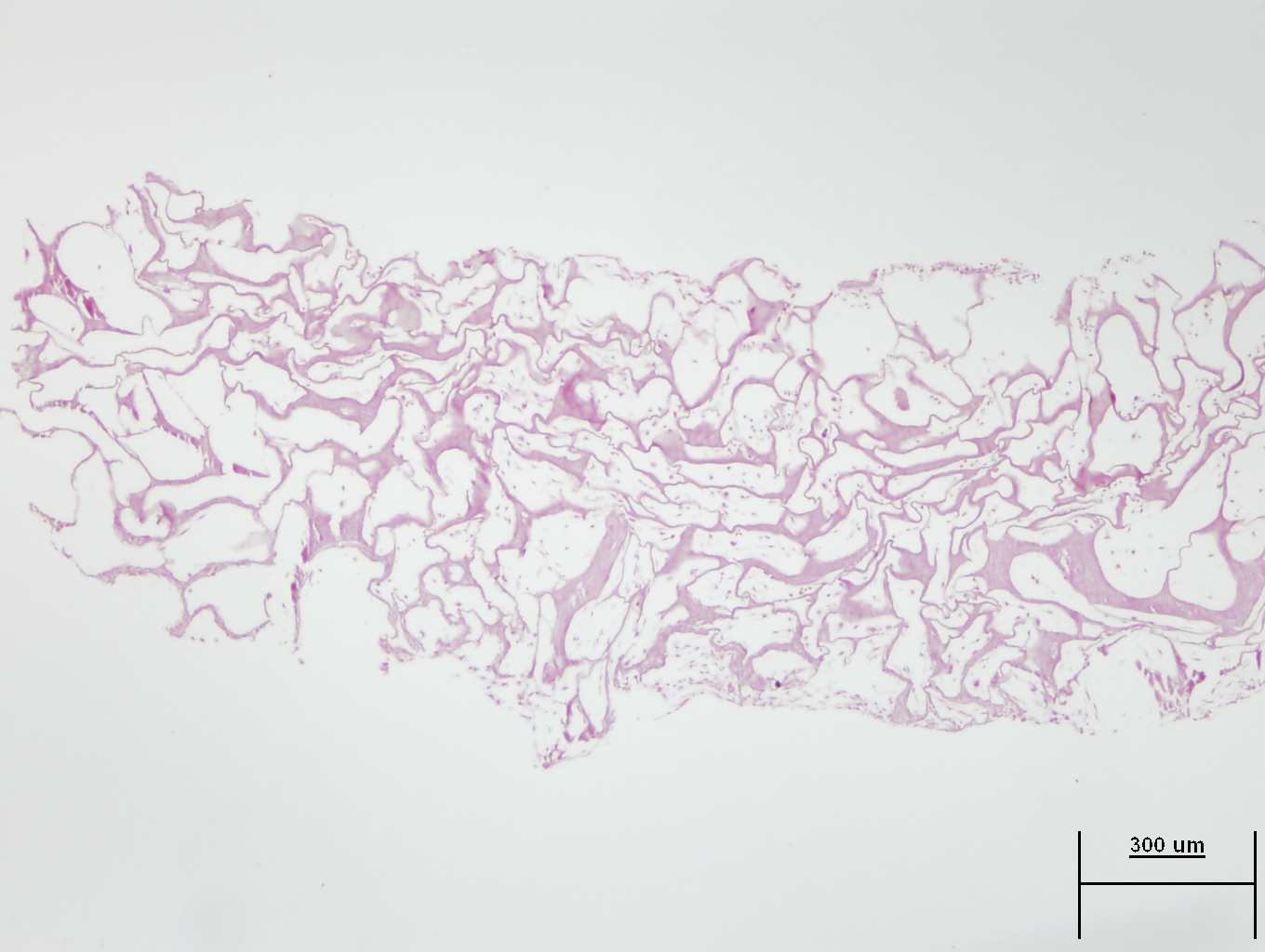

Supplement: Dataset S1 — 1. Description of the folder that has been sent with all the details of the files.2. Comparison between random forest and thresholding. 3. Hyperparameters of the experiment.4. Random Forest validation measurements: Precision, Recall, Confusion Matrix.5. Files to reproduce the experiment (classif and comp folders), including raw data. [file peerj-07-7233-s001.zip › random_forest/base/CCO350.tif]

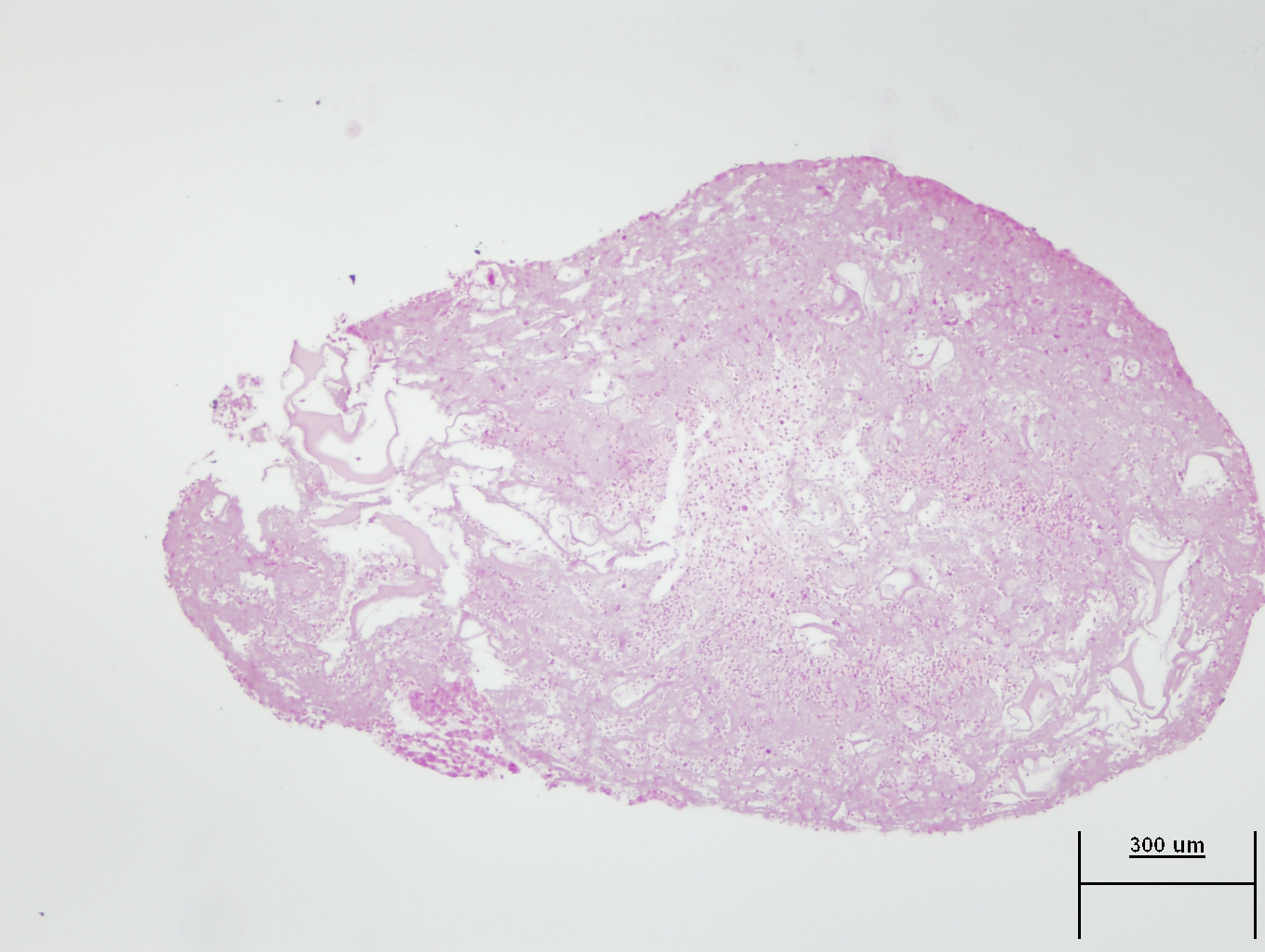

Supplement: Dataset S1 — 1. Description of the folder that has been sent with all the details of the files.2. Comparison between random forest and thresholding. 3. Hyperparameters of the experiment.4. Random Forest validation measurements: Precision, Recall, Confusion Matrix.5. Files to reproduce the experiment (classif and comp folders), including raw data. [file peerj-07-7233-s001.zip › random_forest/base/CCO500.tif]

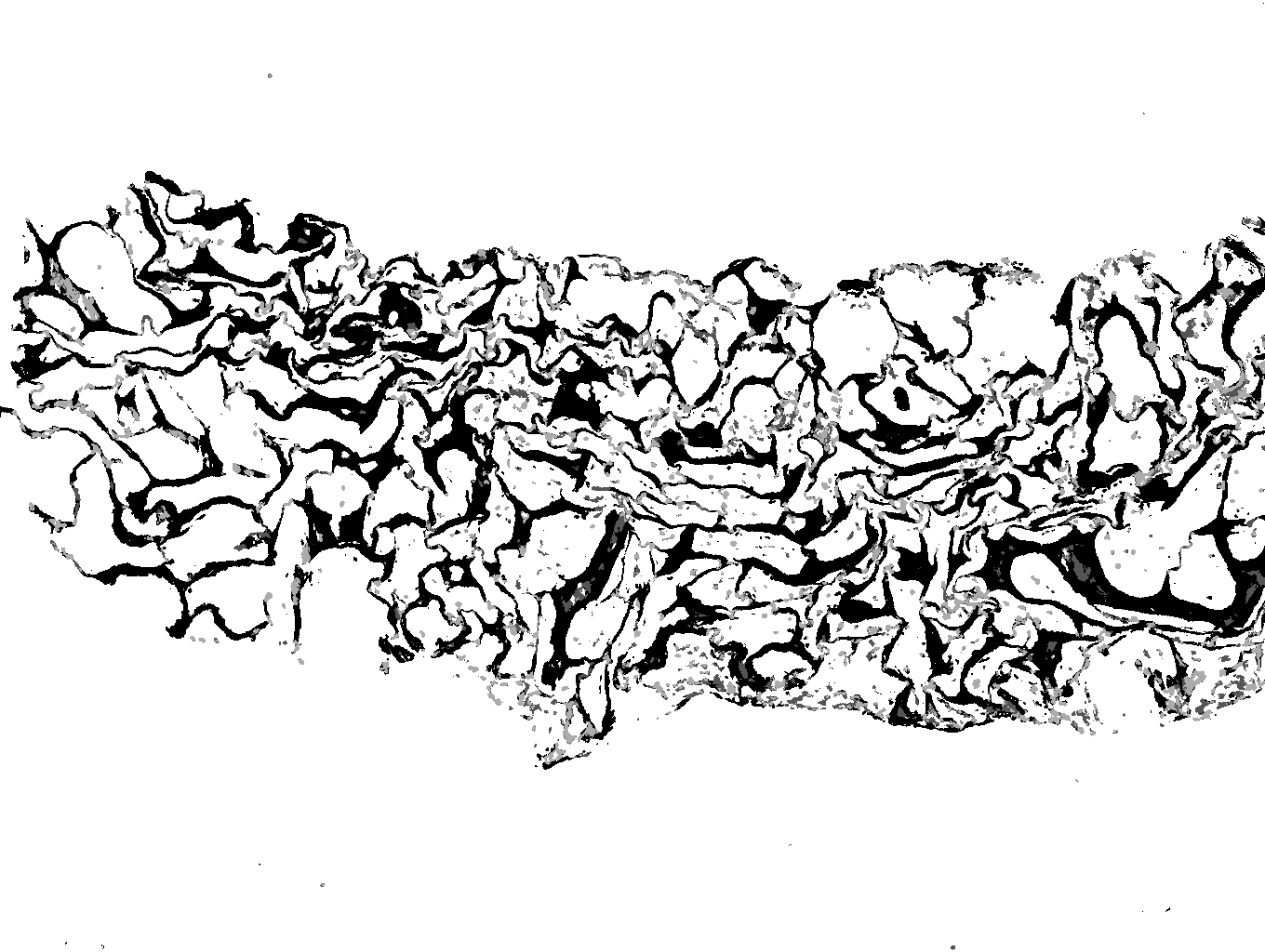

Supplement: Dataset S1 — 1. Description of the folder that has been sent with all the details of the files.2. Comparison between random forest and thresholding. 3. Hyperparameters of the experiment.4. Random Forest validation measurements: Precision, Recall, Confusion Matrix.5. Files to reproduce the experiment (classif and comp folders), including raw data. [file peerj-07-7233-s001.zip › random_forest/CCO350_I_classified.png]

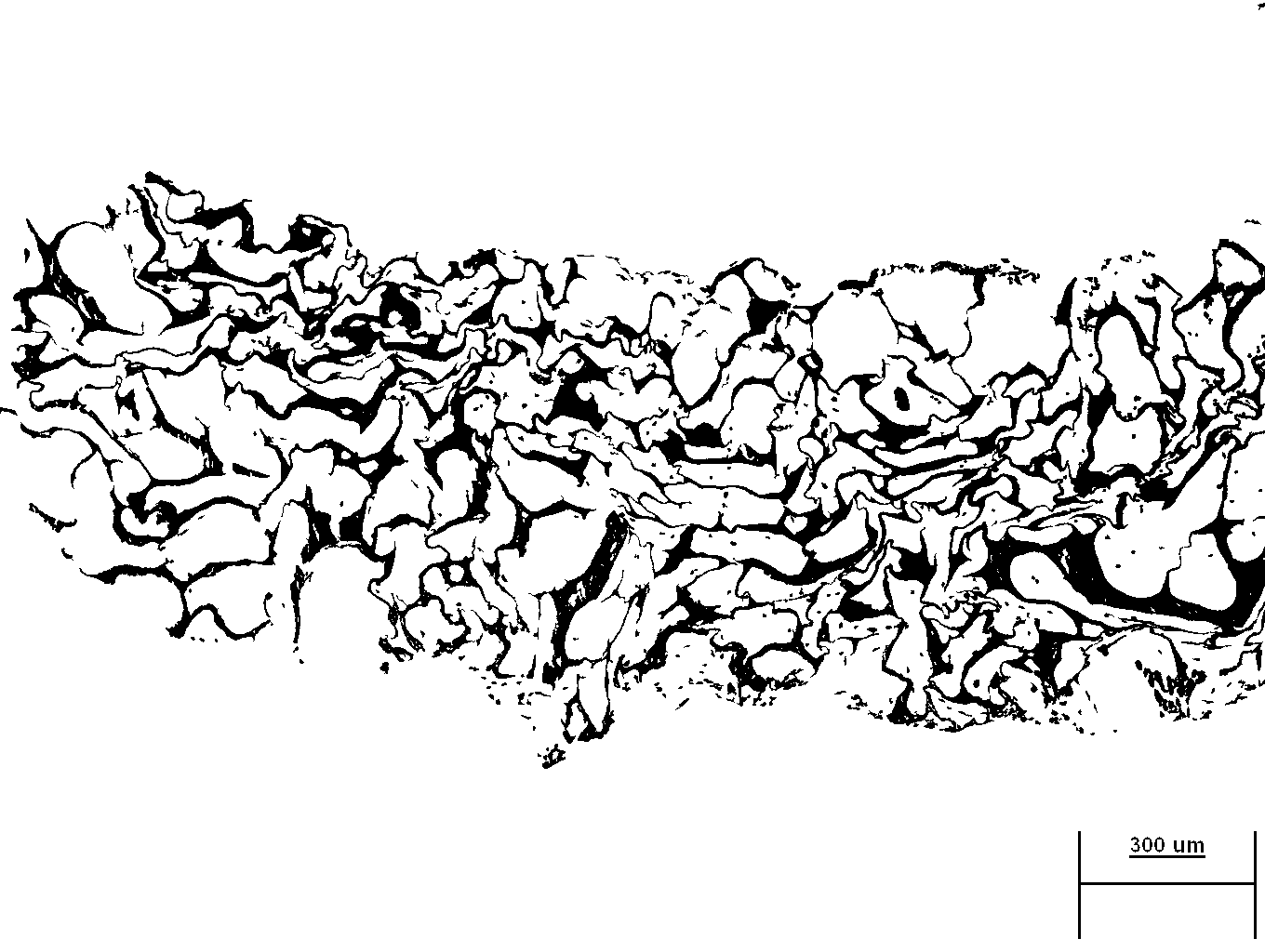

Supplement: Dataset S1 — 1. Description of the folder that has been sent with all the details of the files.2. Comparison between random forest and thresholding. 3. Hyperparameters of the experiment.4. Random Forest validation measurements: Precision, Recall, Confusion Matrix.5. Files to reproduce the experiment (classif and comp folders), including raw data. [file peerj-07-7233-s001.zip › random_forest/CCO350_thresh-triangle_size10-inf.tif]

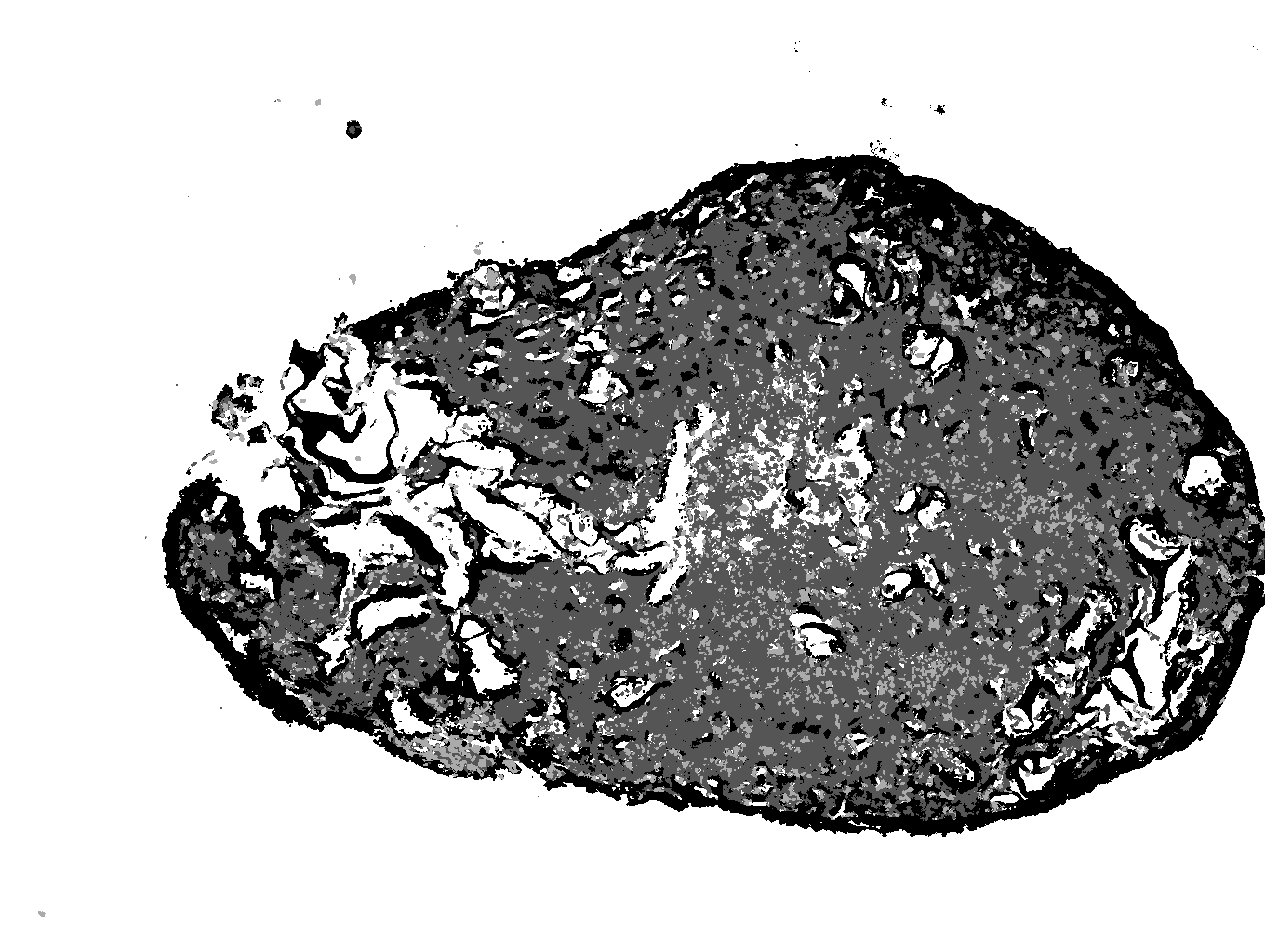

Supplement: Dataset S1 — 1. Description of the folder that has been sent with all the details of the files.2. Comparison between random forest and thresholding. 3. Hyperparameters of the experiment.4. Random Forest validation measurements: Precision, Recall, Confusion Matrix.5. Files to reproduce the experiment (classif and comp folders), including raw data. [file peerj-07-7233-s001.zip › random_forest/CCO500_I_classified.png]

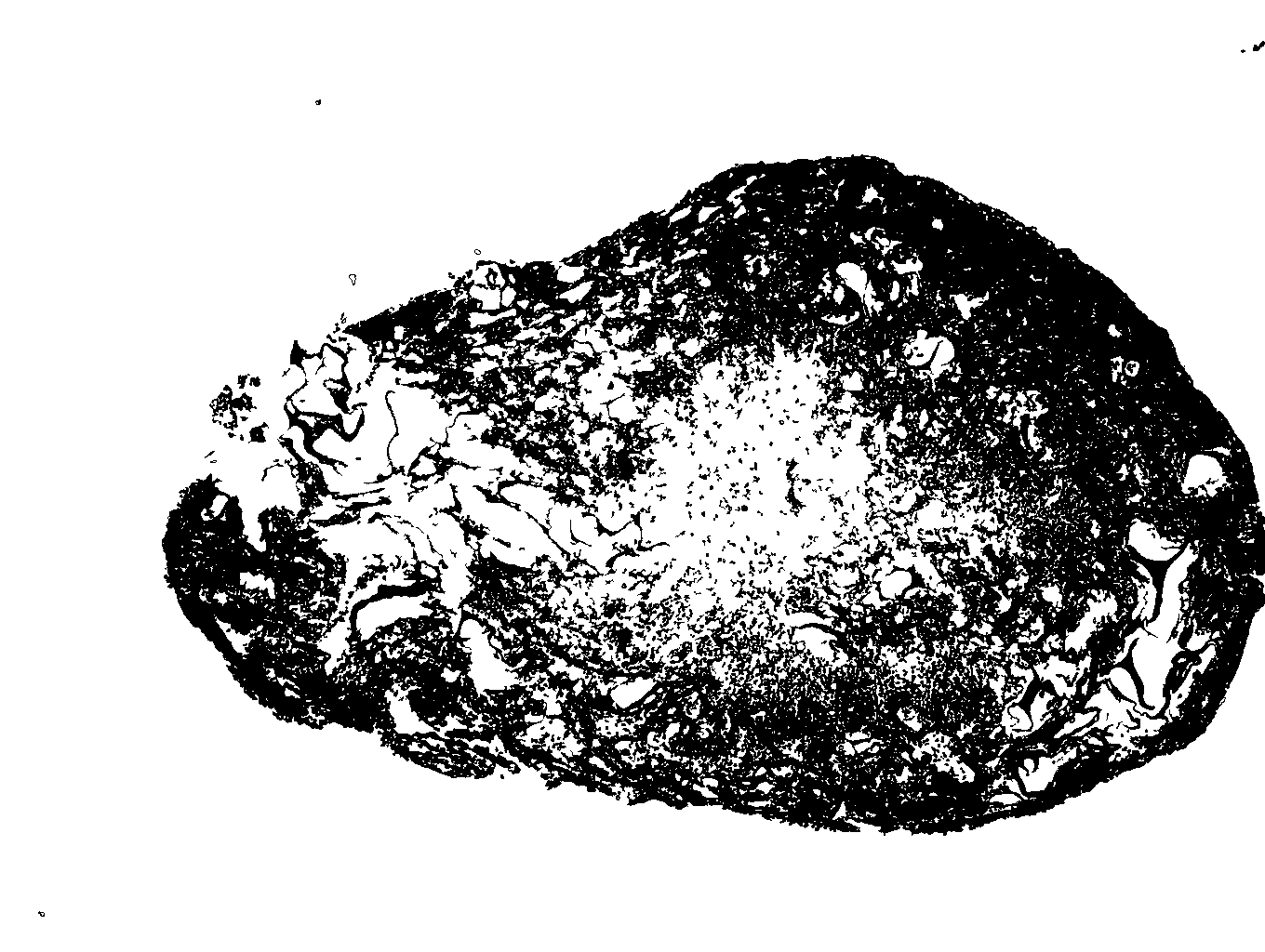

Supplement: Dataset S1 — 1. Description of the folder that has been sent with all the details of the files.2. Comparison between random forest and thresholding. 3. Hyperparameters of the experiment.4. Random Forest validation measurements: Precision, Recall, Confusion Matrix.5. Files to reproduce the experiment (classif and comp folders), including raw data. [file peerj-07-7233-s001.zip › random_forest/CCO500_thresh-manual_size10-inf.tif]

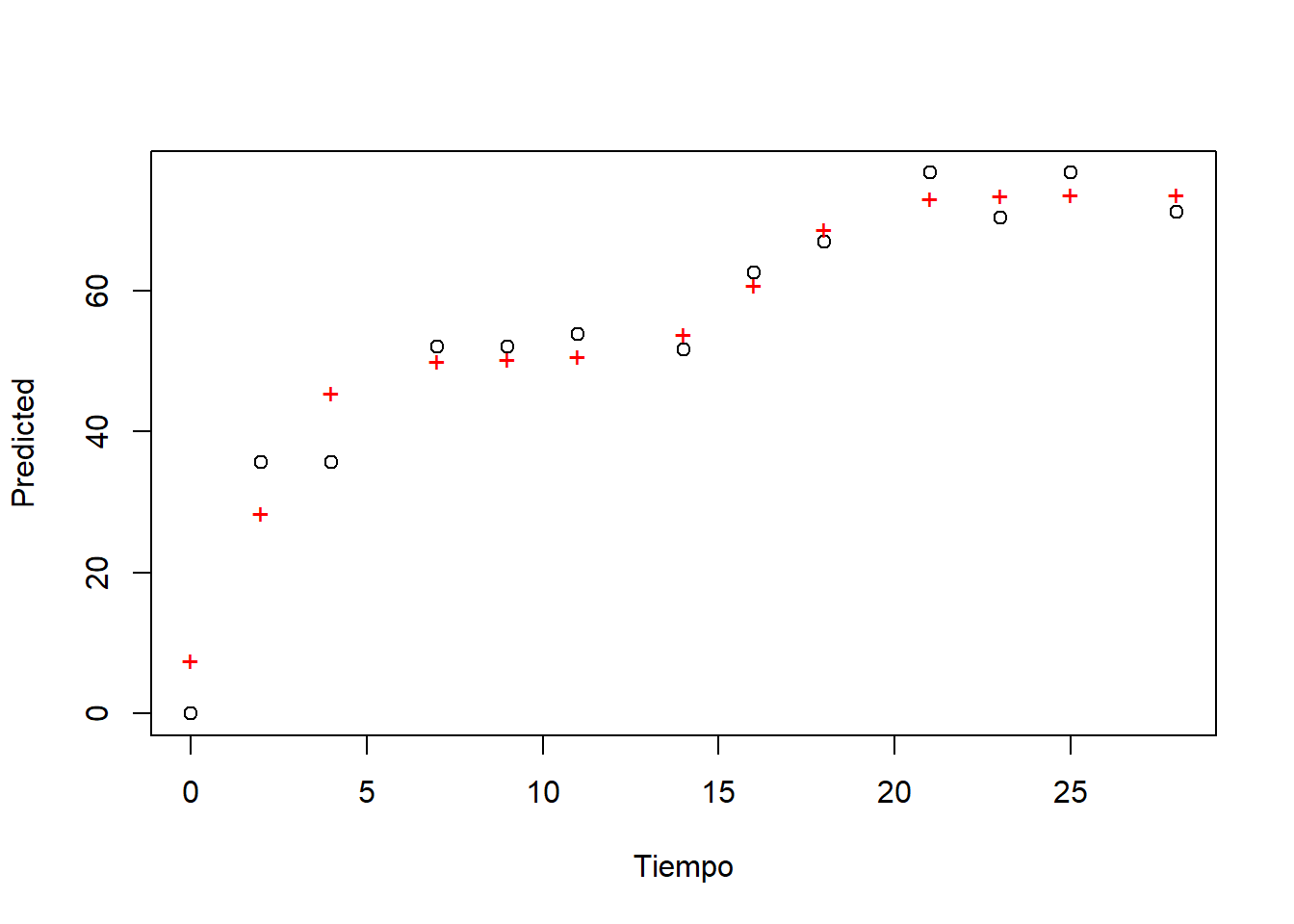

Supplement: Supplemental Information 1 — A html report is developed with R markdown and included with the corresponding files. Code and outputs are included. [file peerj-07-7233-s004.zip › Scripts_and_Outputs_files/figure-html/unnamed-chunk-10-1.png]

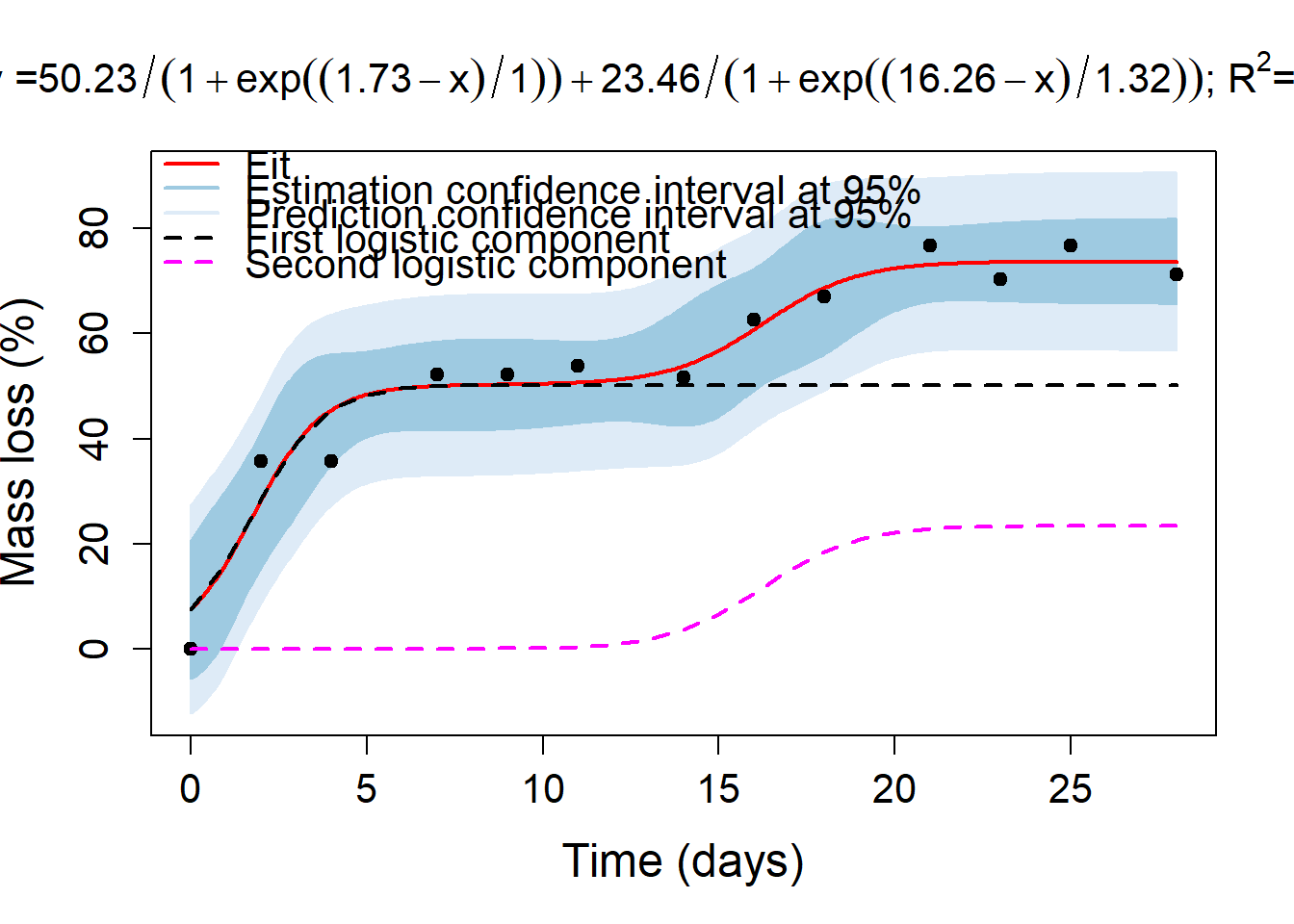

Supplement: Supplemental Information 1 — A html report is developed with R markdown and included with the corresponding files. Code and outputs are included. [file peerj-07-7233-s004.zip › Scripts_and_Outputs_files/figure-html/unnamed-chunk-12-1.png]

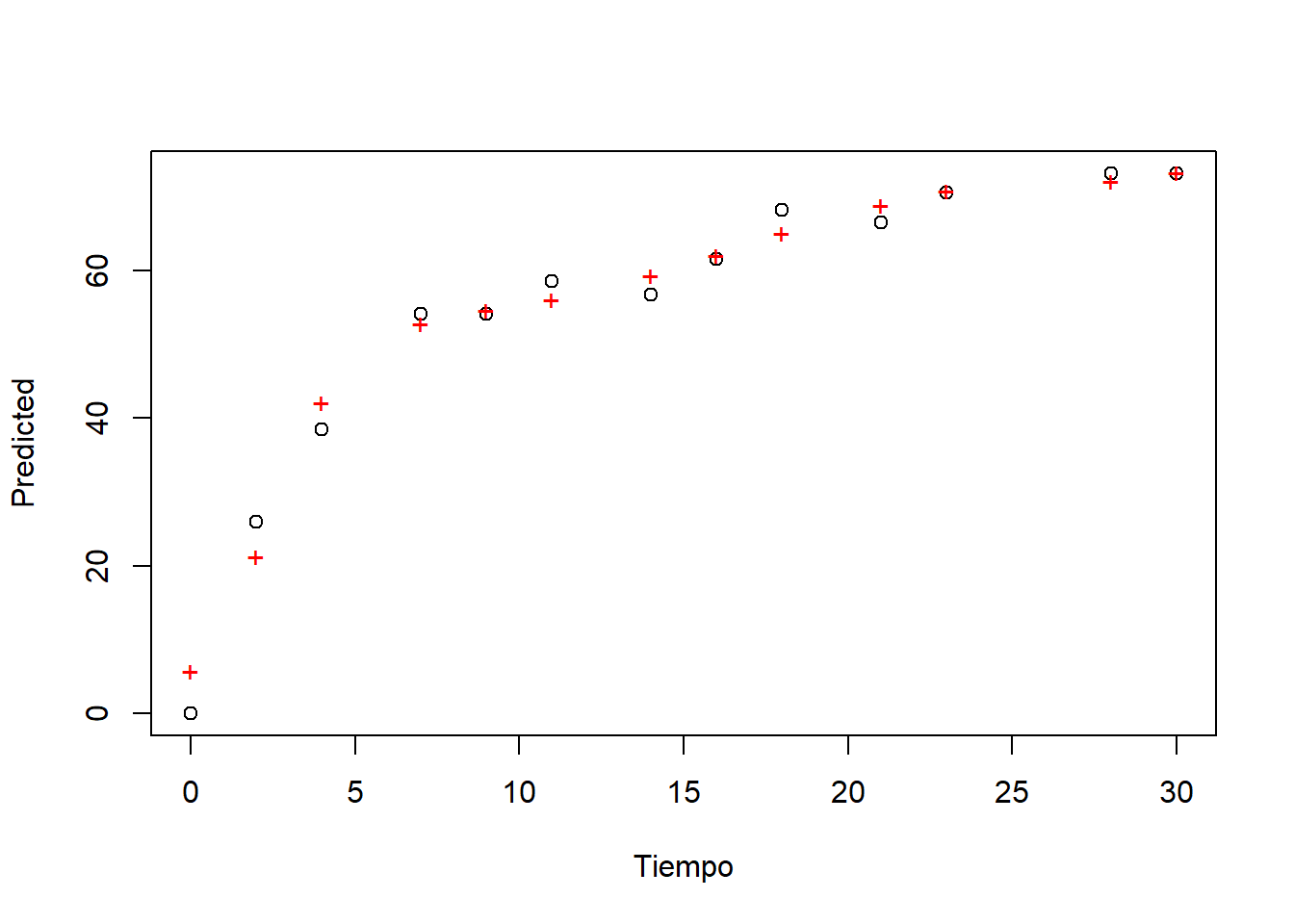

Supplement: Supplemental Information 1 — A html report is developed with R markdown and included with the corresponding files. Code and outputs are included. [file peerj-07-7233-s004.zip › Scripts_and_Outputs_files/figure-html/unnamed-chunk-15-1.png]

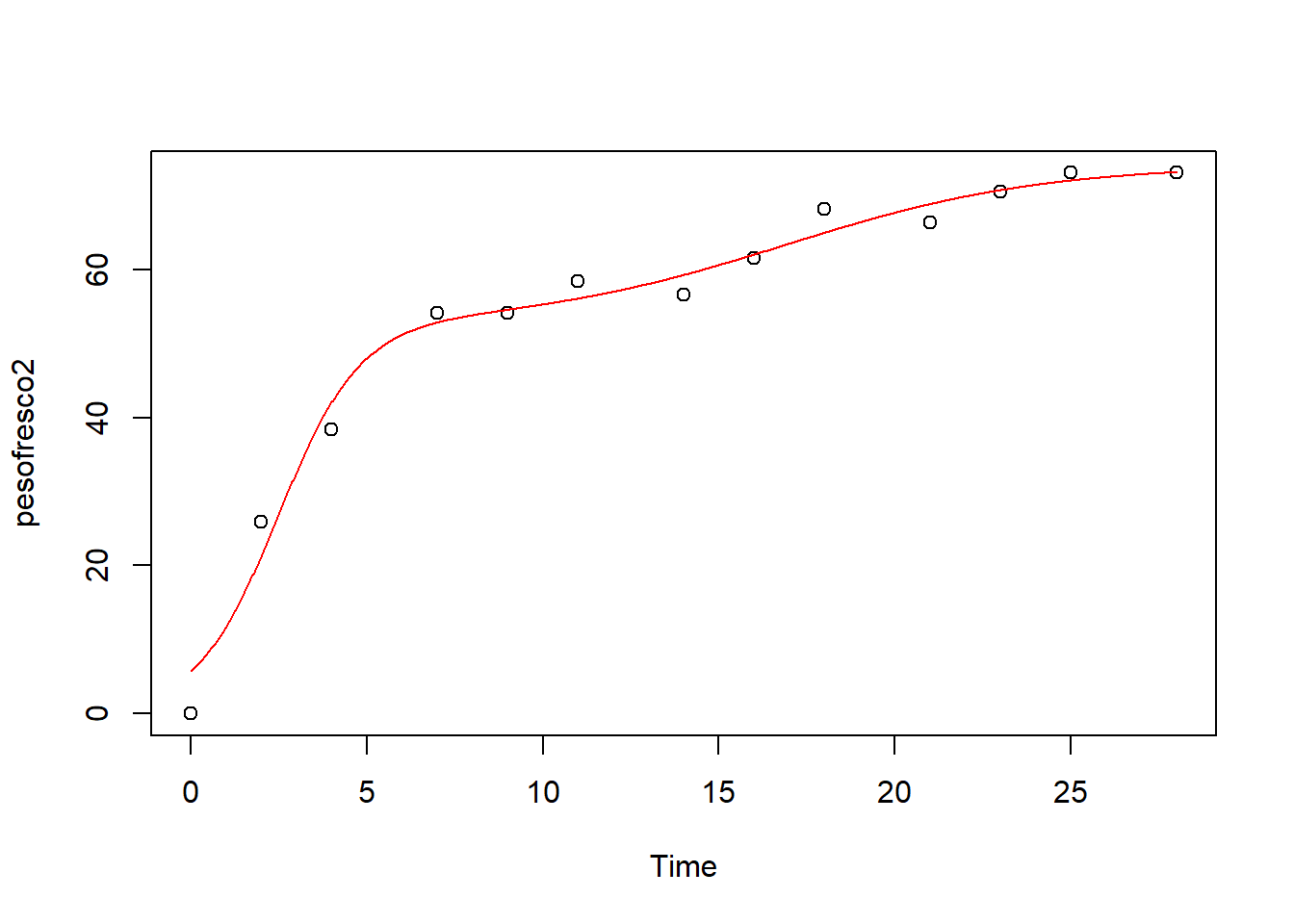

Supplement: Supplemental Information 1 — A html report is developed with R markdown and included with the corresponding files. Code and outputs are included. [file peerj-07-7233-s004.zip › Scripts_and_Outputs_files/figure-html/unnamed-chunk-17-1.png]

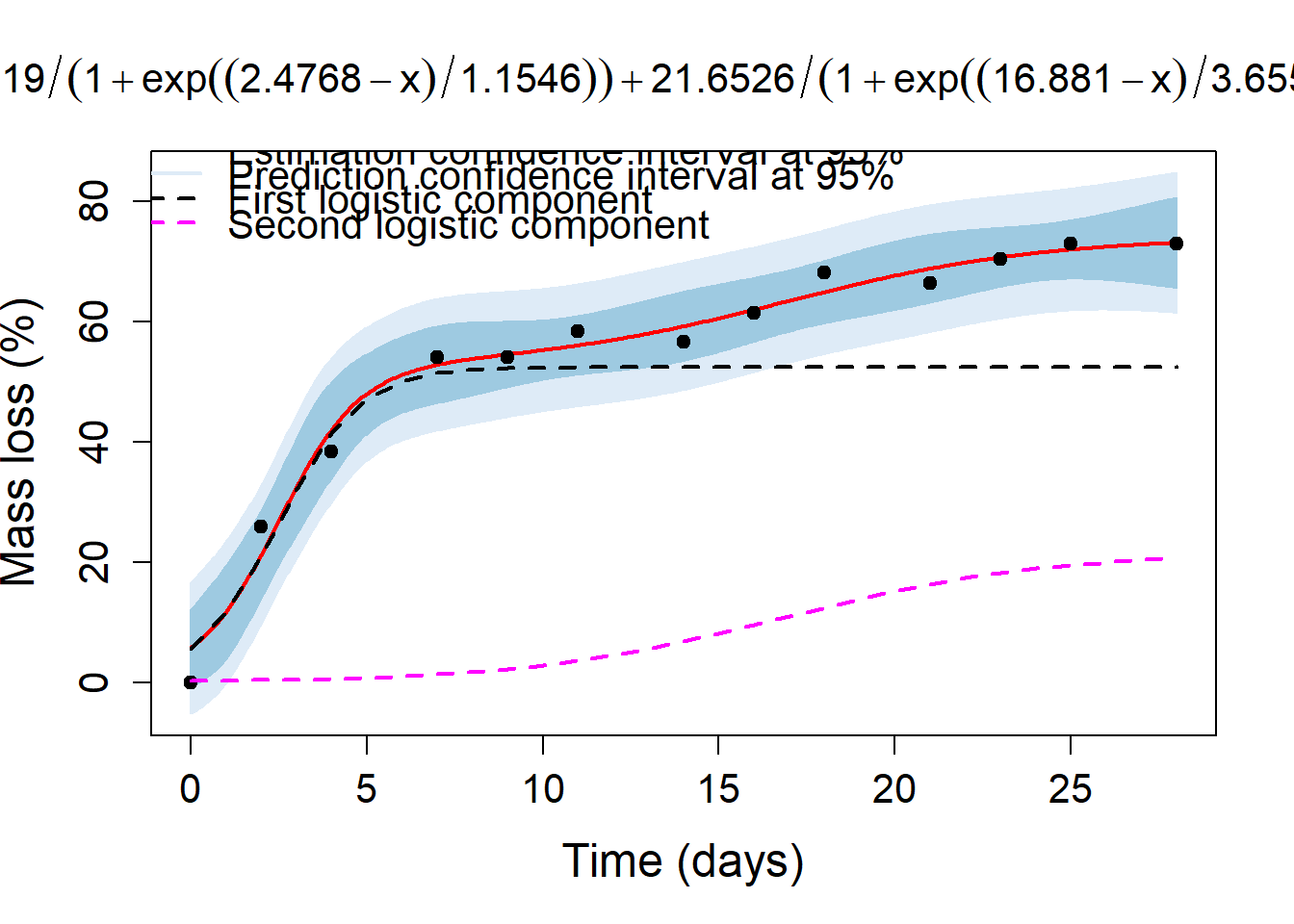

Supplement: Supplemental Information 1 — A html report is developed with R markdown and included with the corresponding files. Code and outputs are included. [file peerj-07-7233-s004.zip › Scripts_and_Outputs_files/figure-html/unnamed-chunk-18-1.png]

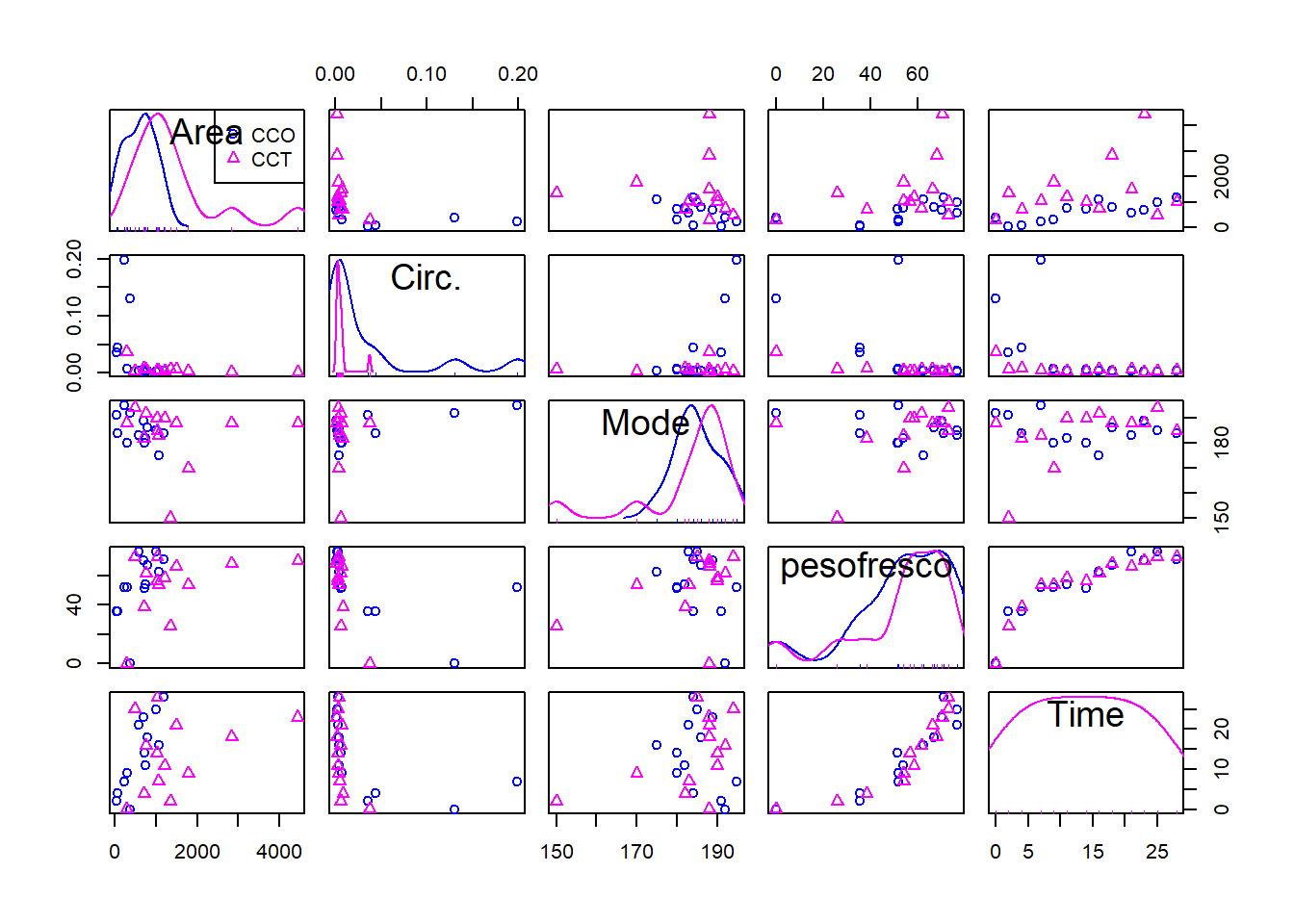

Supplement: Supplemental Information 1 — A html report is developed with R markdown and included with the corresponding files. Code and outputs are included. [file peerj-07-7233-s004.zip › Scripts_and_Outputs_files/figure-html/unnamed-chunk-2-1.png]

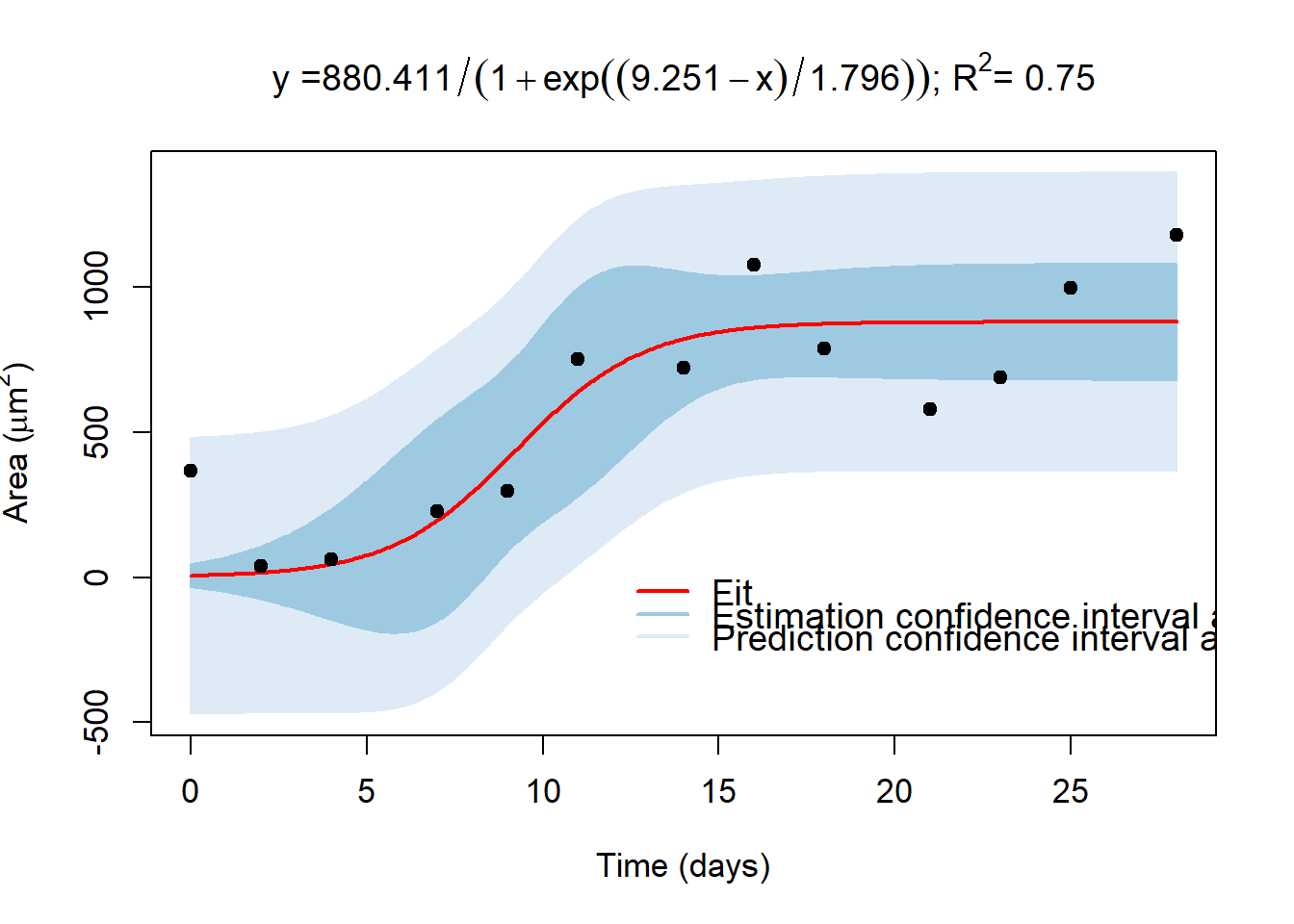

Supplement: Supplemental Information 1 — A html report is developed with R markdown and included with the corresponding files. Code and outputs are included. [file peerj-07-7233-s004.zip › Scripts_and_Outputs_files/figure-html/unnamed-chunk-21-1.png]

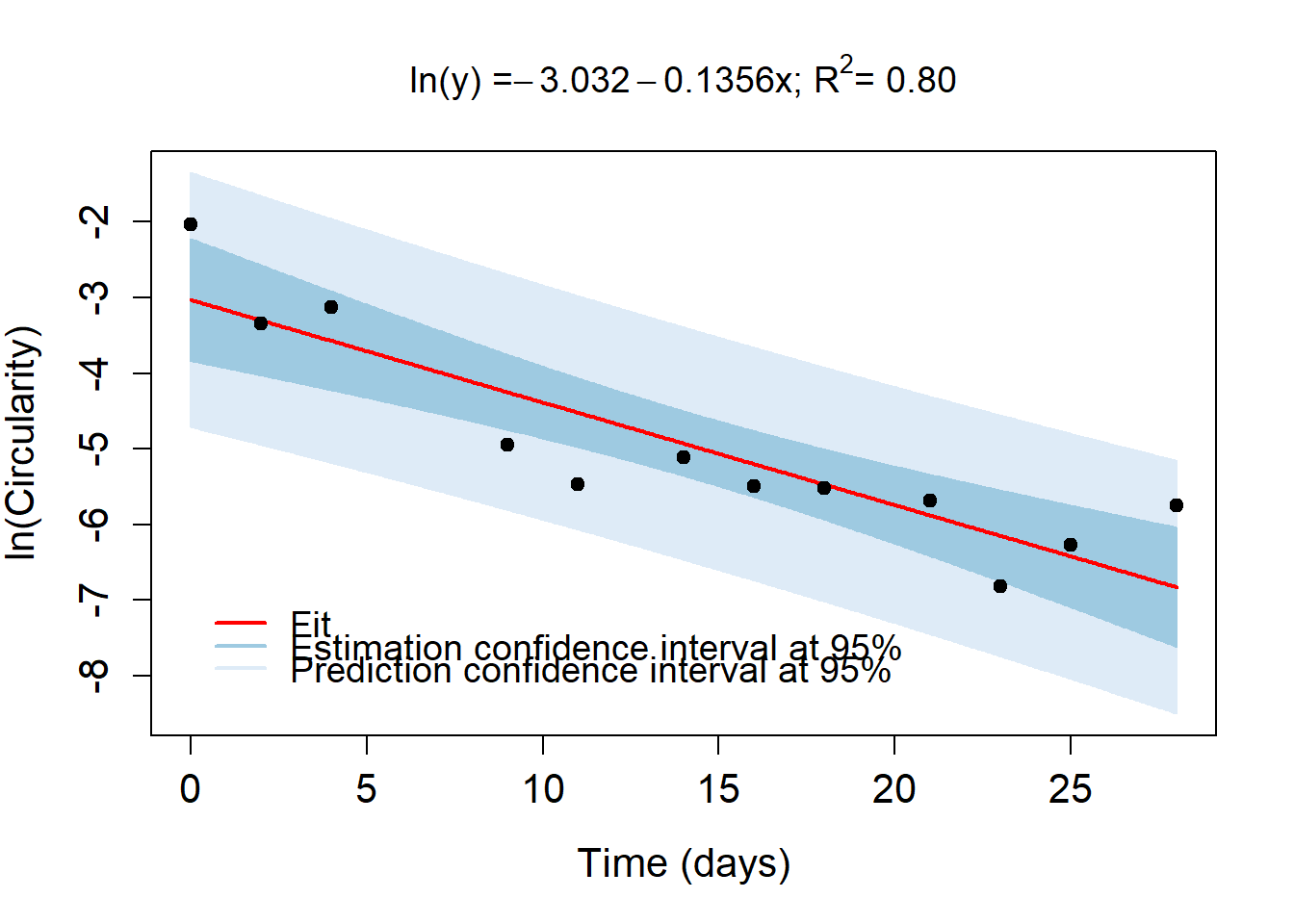

Supplement: Supplemental Information 1 — A html report is developed with R markdown and included with the corresponding files. Code and outputs are included. [file peerj-07-7233-s004.zip › Scripts_and_Outputs_files/figure-html/unnamed-chunk-22-1.png]

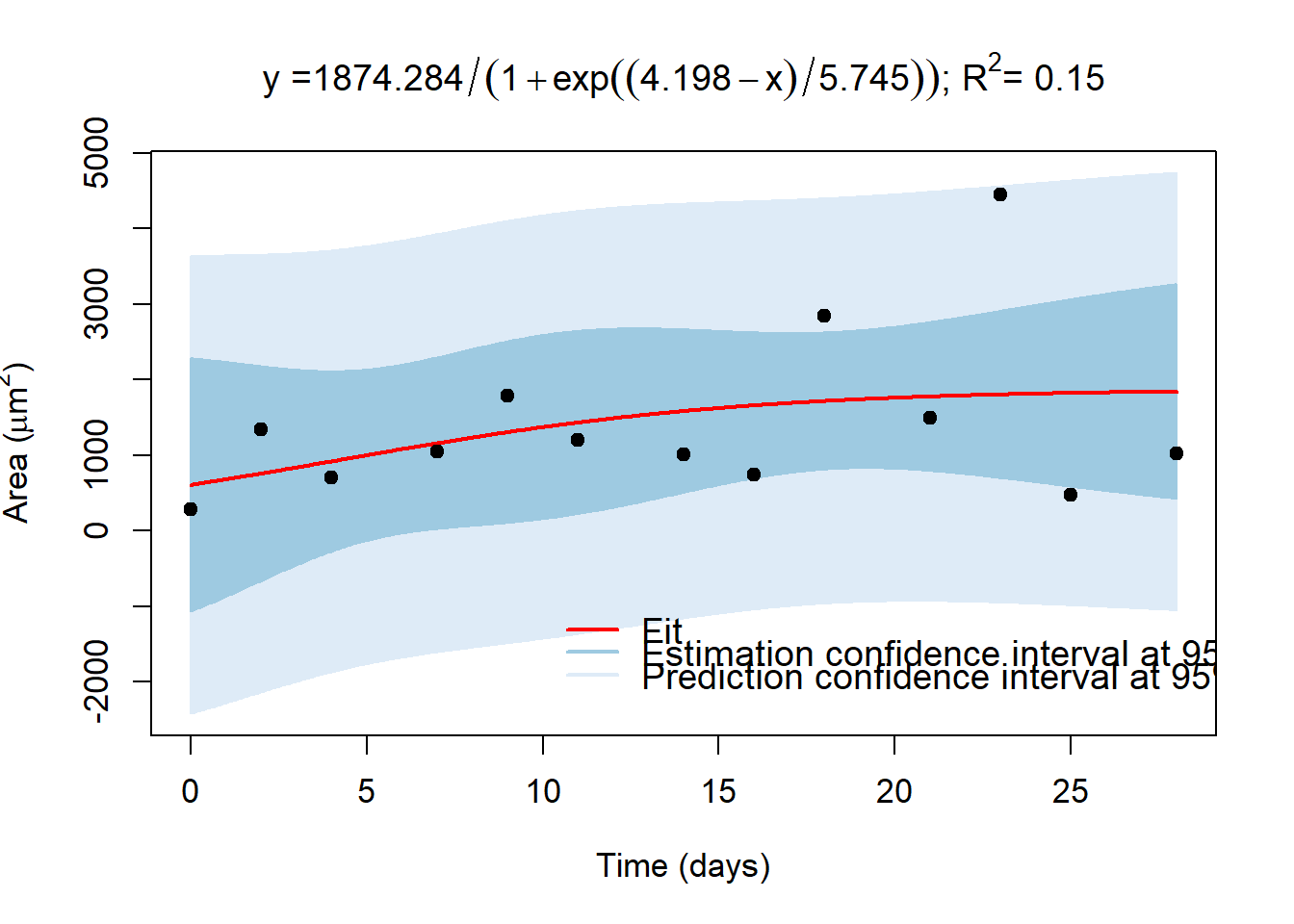

Supplement: Supplemental Information 1 — A html report is developed with R markdown and included with the corresponding files. Code and outputs are included. [file peerj-07-7233-s004.zip › Scripts_and_Outputs_files/figure-html/unnamed-chunk-24-1.png]

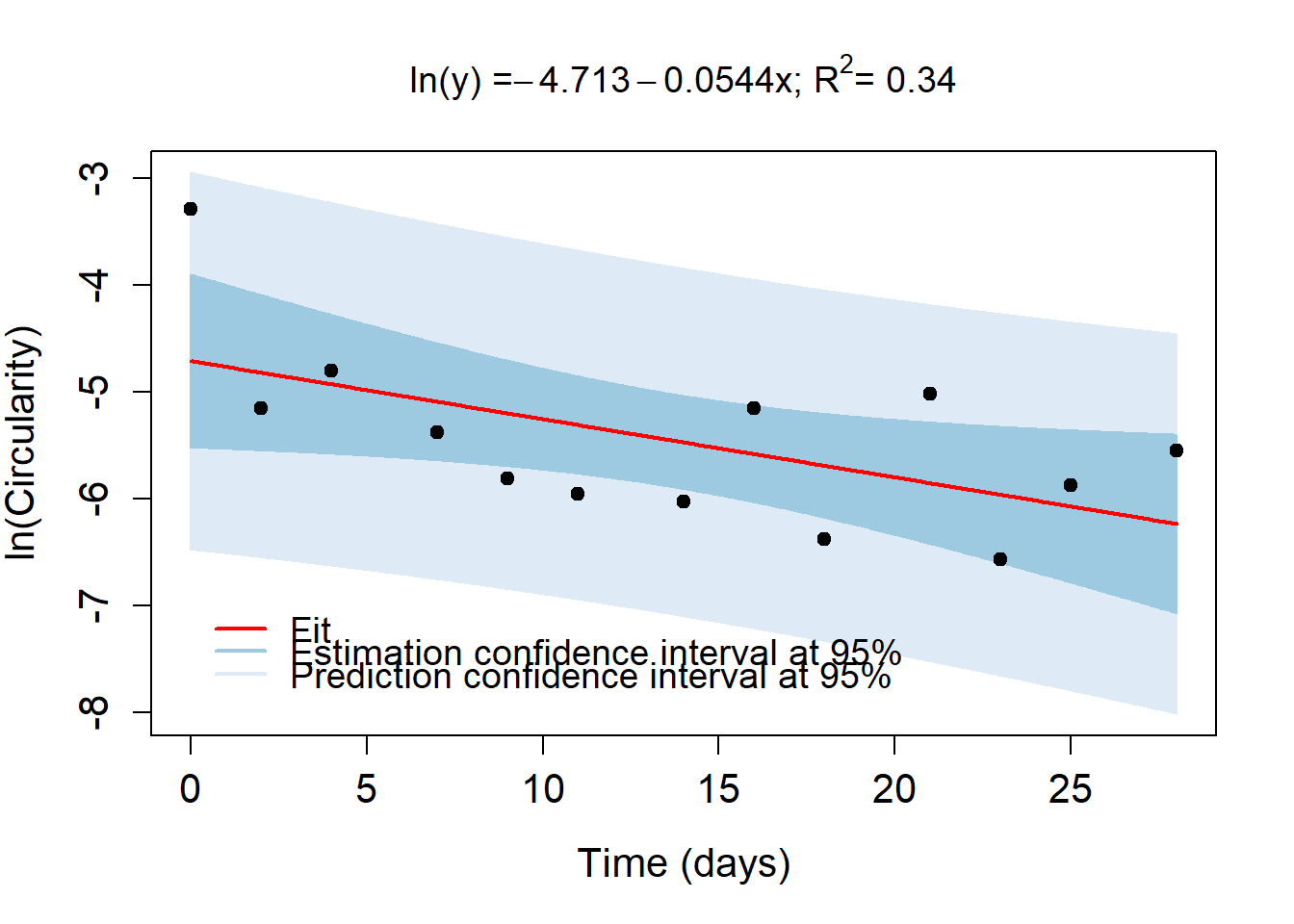

Supplement: Supplemental Information 1 — A html report is developed with R markdown and included with the corresponding files. Code and outputs are included. [file peerj-07-7233-s004.zip › Scripts_and_Outputs_files/figure-html/unnamed-chunk-25-1.png]

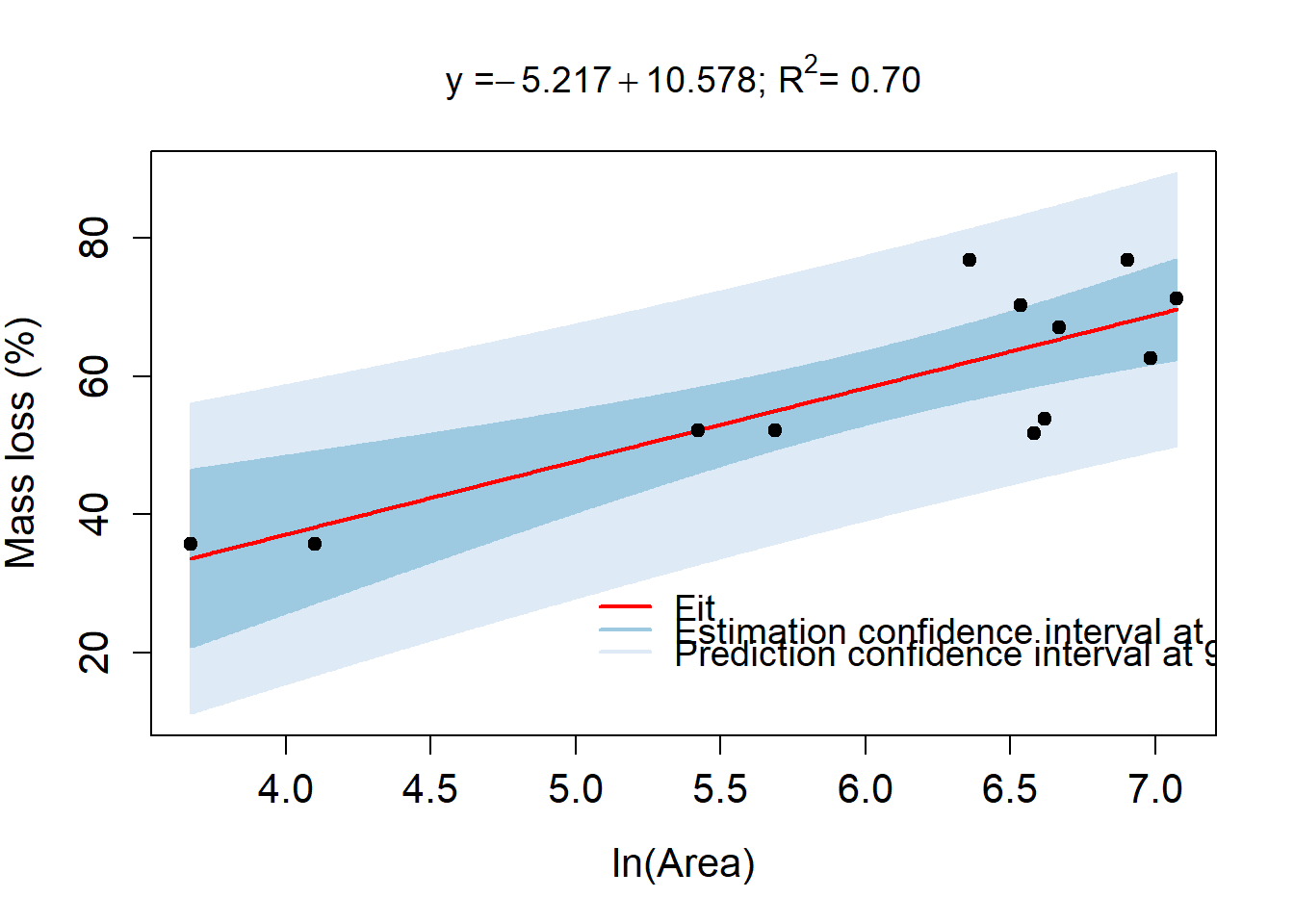

Supplement: Supplemental Information 1 — A html report is developed with R markdown and included with the corresponding files. Code and outputs are included. [file peerj-07-7233-s004.zip › Scripts_and_Outputs_files/figure-html/unnamed-chunk-26-1.png]

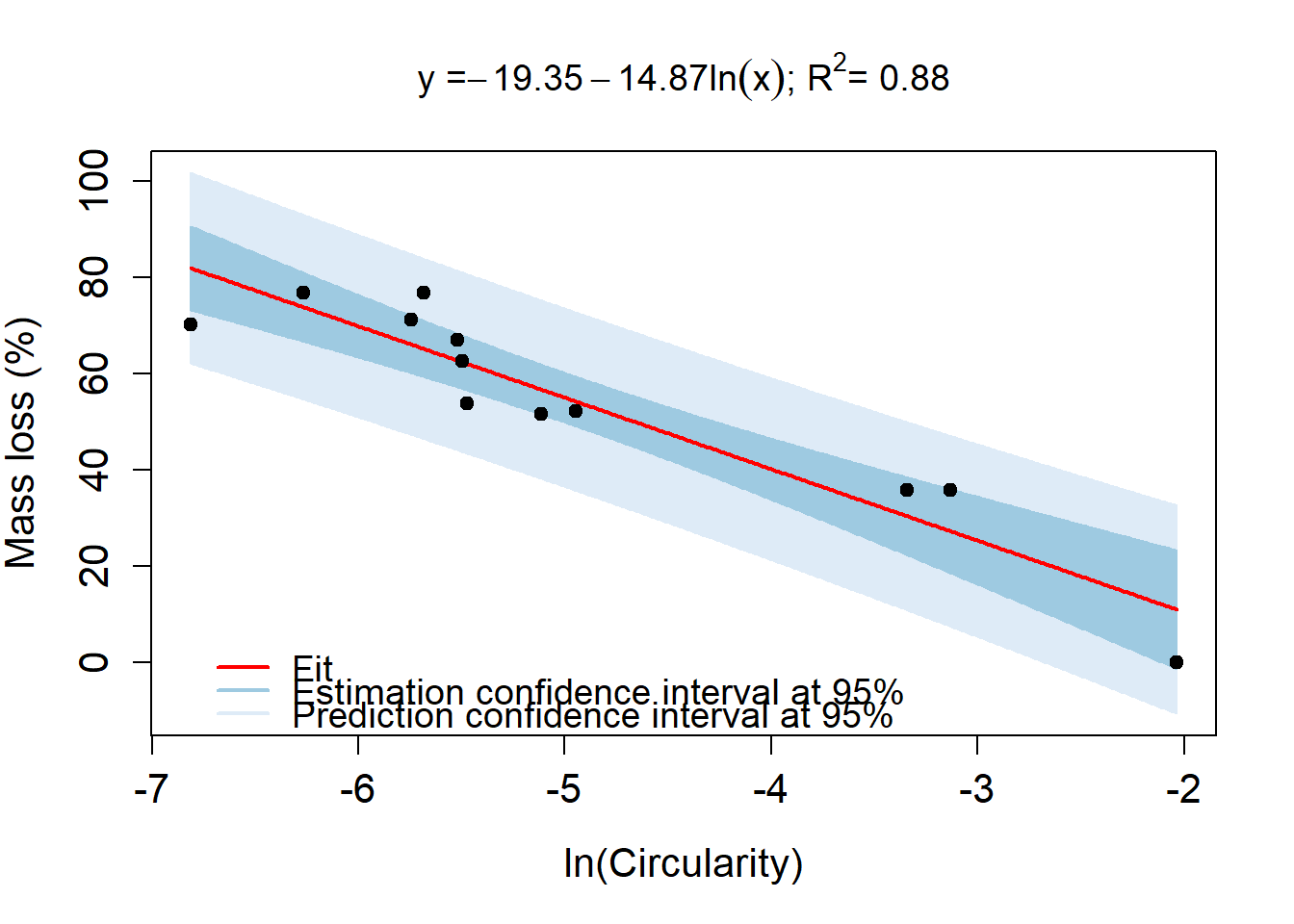

Supplement: Supplemental Information 1 — A html report is developed with R markdown and included with the corresponding files. Code and outputs are included. [file peerj-07-7233-s004.zip › Scripts_and_Outputs_files/figure-html/unnamed-chunk-27-1.png]

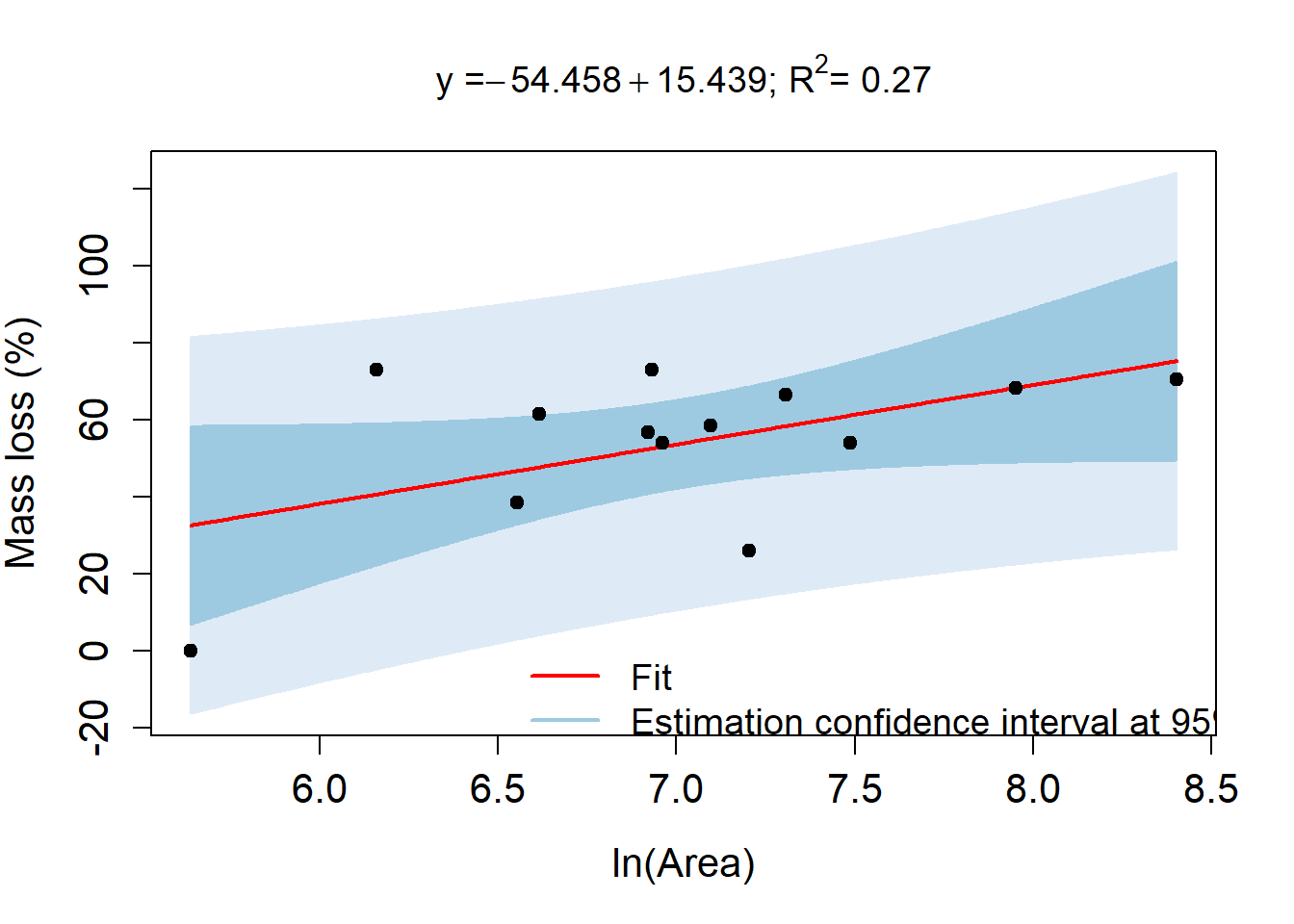

Supplement: Supplemental Information 1 — A html report is developed with R markdown and included with the corresponding files. Code and outputs are included. [file peerj-07-7233-s004.zip › Scripts_and_Outputs_files/figure-html/unnamed-chunk-28-1.png]

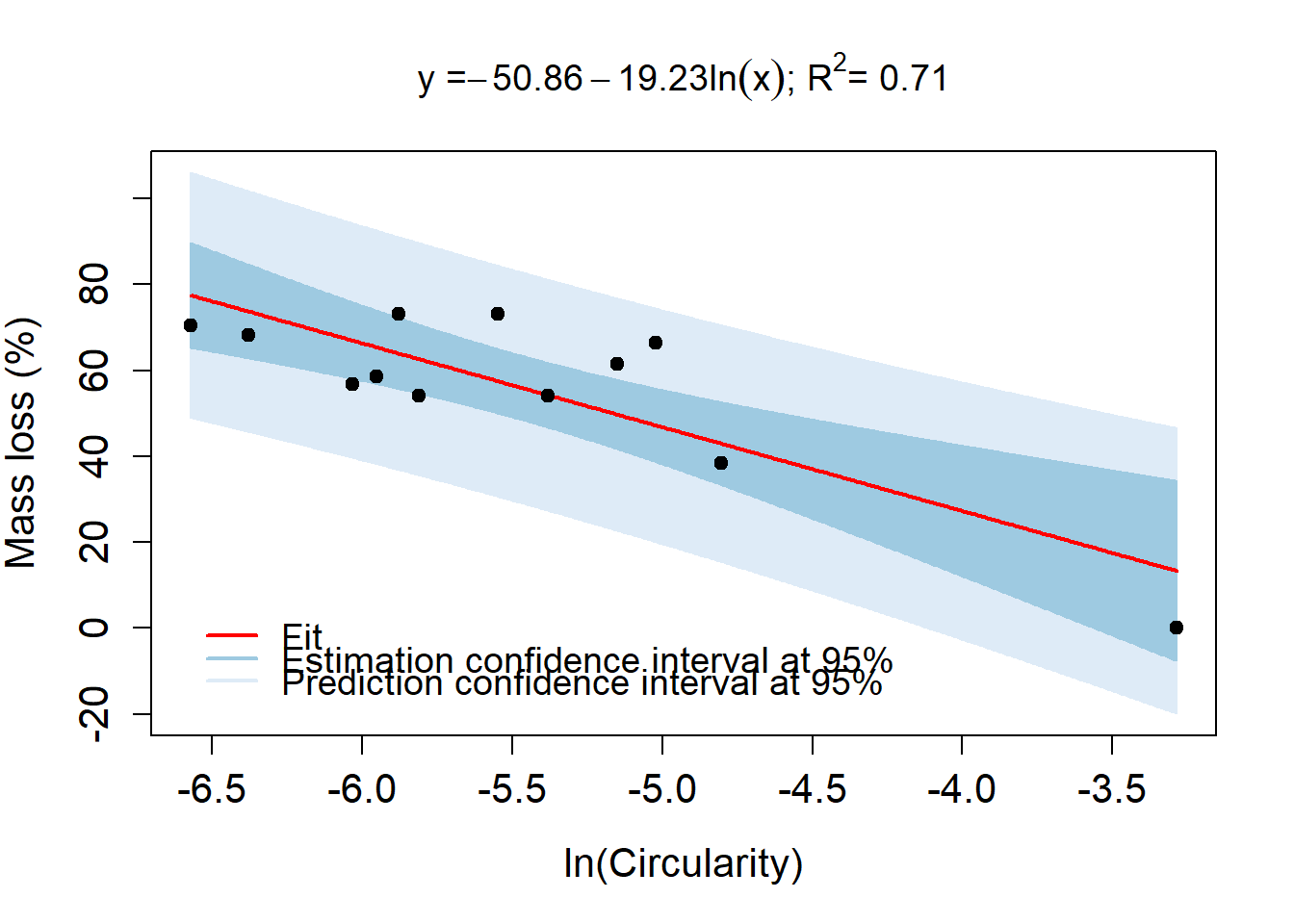

Supplement: Supplemental Information 1 — A html report is developed with R markdown and included with the corresponding files. Code and outputs are included. [file peerj-07-7233-s004.zip › Scripts_and_Outputs_files/figure-html/unnamed-chunk-29-1.png]

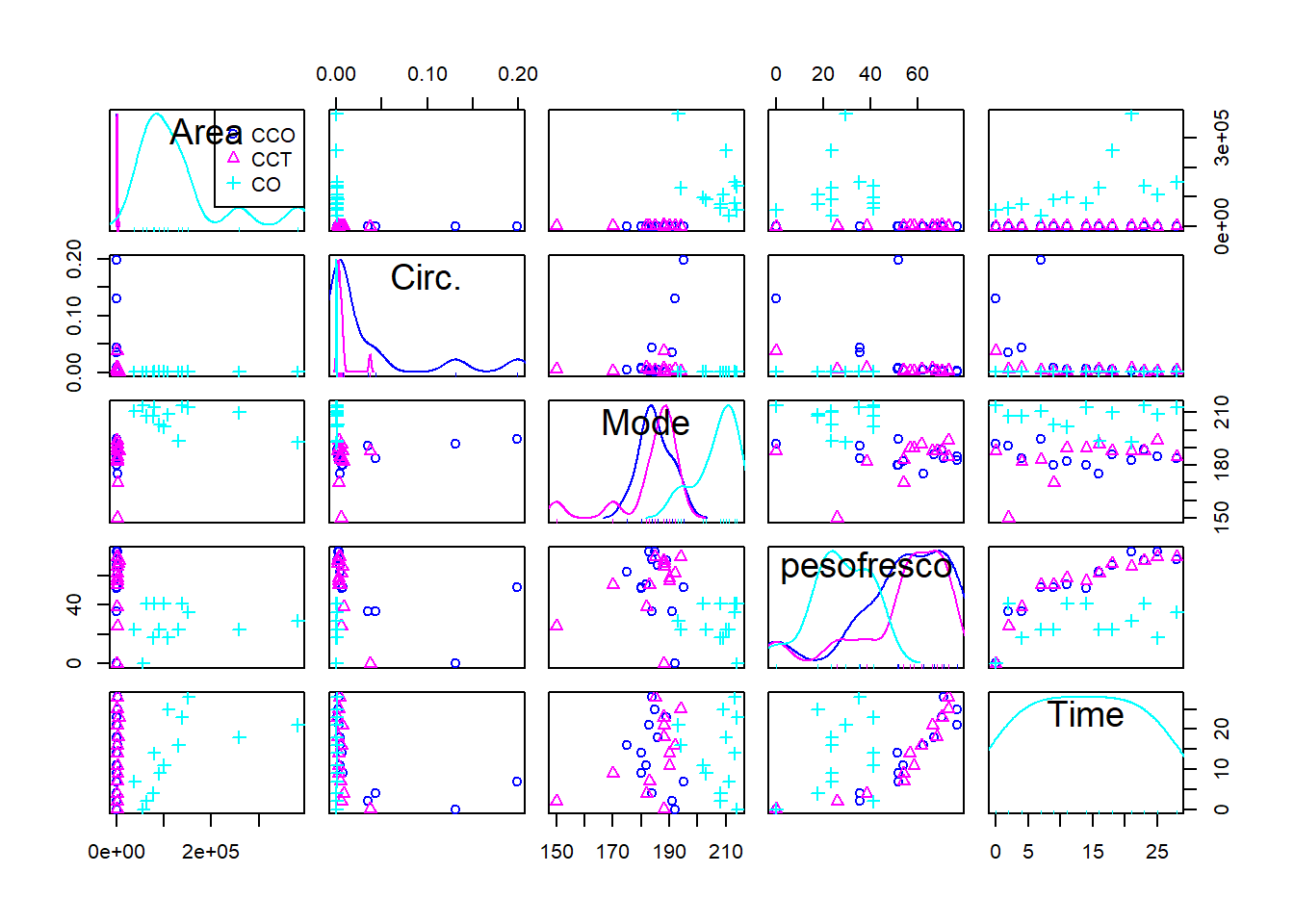

Supplement: Supplemental Information 1 — A html report is developed with R markdown and included with the corresponding files. Code and outputs are included. [file peerj-07-7233-s004.zip › Scripts_and_Outputs_files/figure-html/unnamed-chunk-3-1.png]

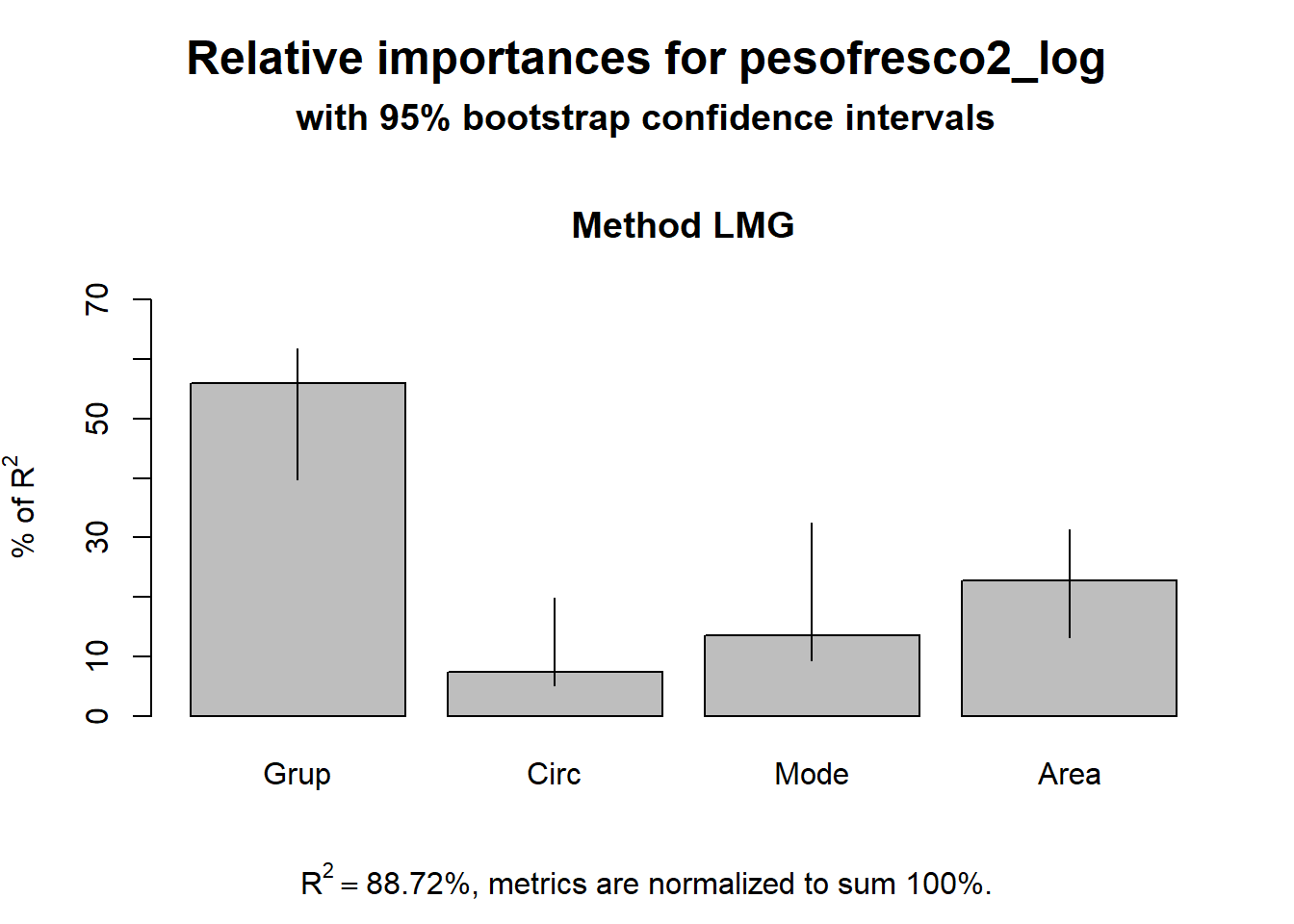

Supplement: Supplemental Information 1 — A html report is developed with R markdown and included with the corresponding files. Code and outputs are included. [file peerj-07-7233-s004.zip › Scripts_and_Outputs_files/figure-html/unnamed-chunk-33-1.png]

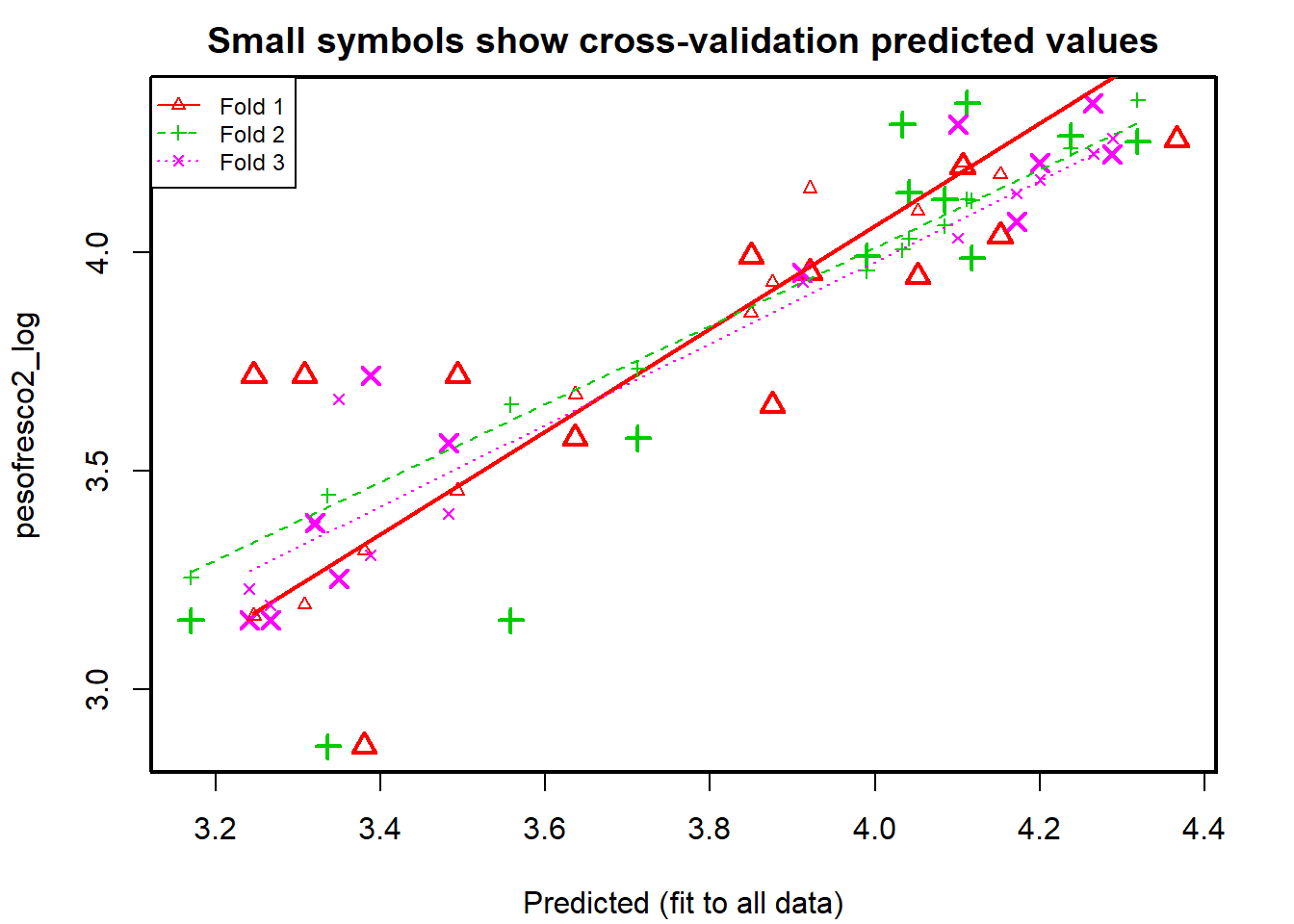

Supplement: Supplemental Information 1 — A html report is developed with R markdown and included with the corresponding files. Code and outputs are included. [file peerj-07-7233-s004.zip › Scripts_and_Outputs_files/figure-html/unnamed-chunk-34-1.png]

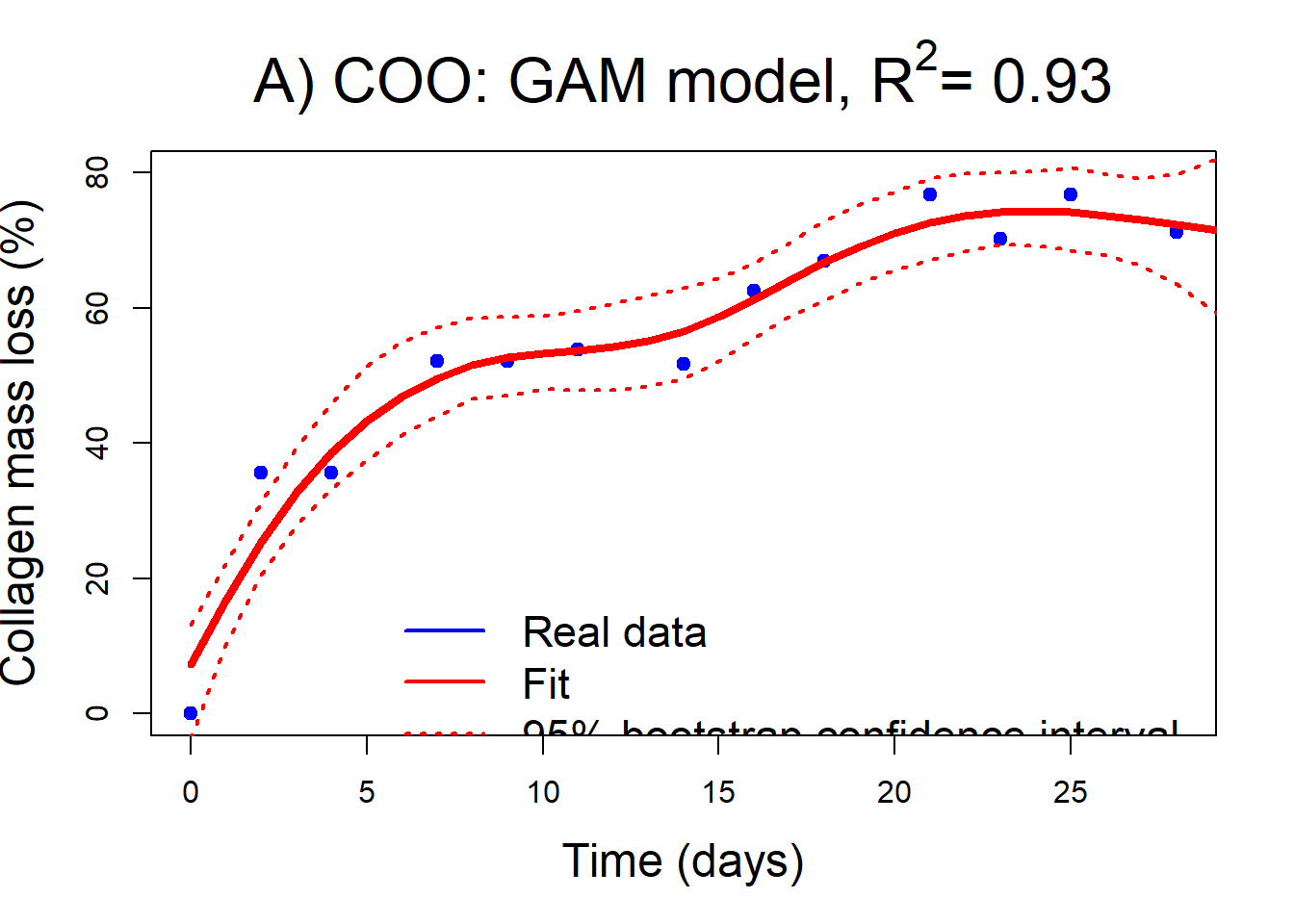

Supplement: Supplemental Information 1 — A html report is developed with R markdown and included with the corresponding files. Code and outputs are included. [file peerj-07-7233-s004.zip › Scripts_and_Outputs_files/figure-html/unnamed-chunk-5-1.png]

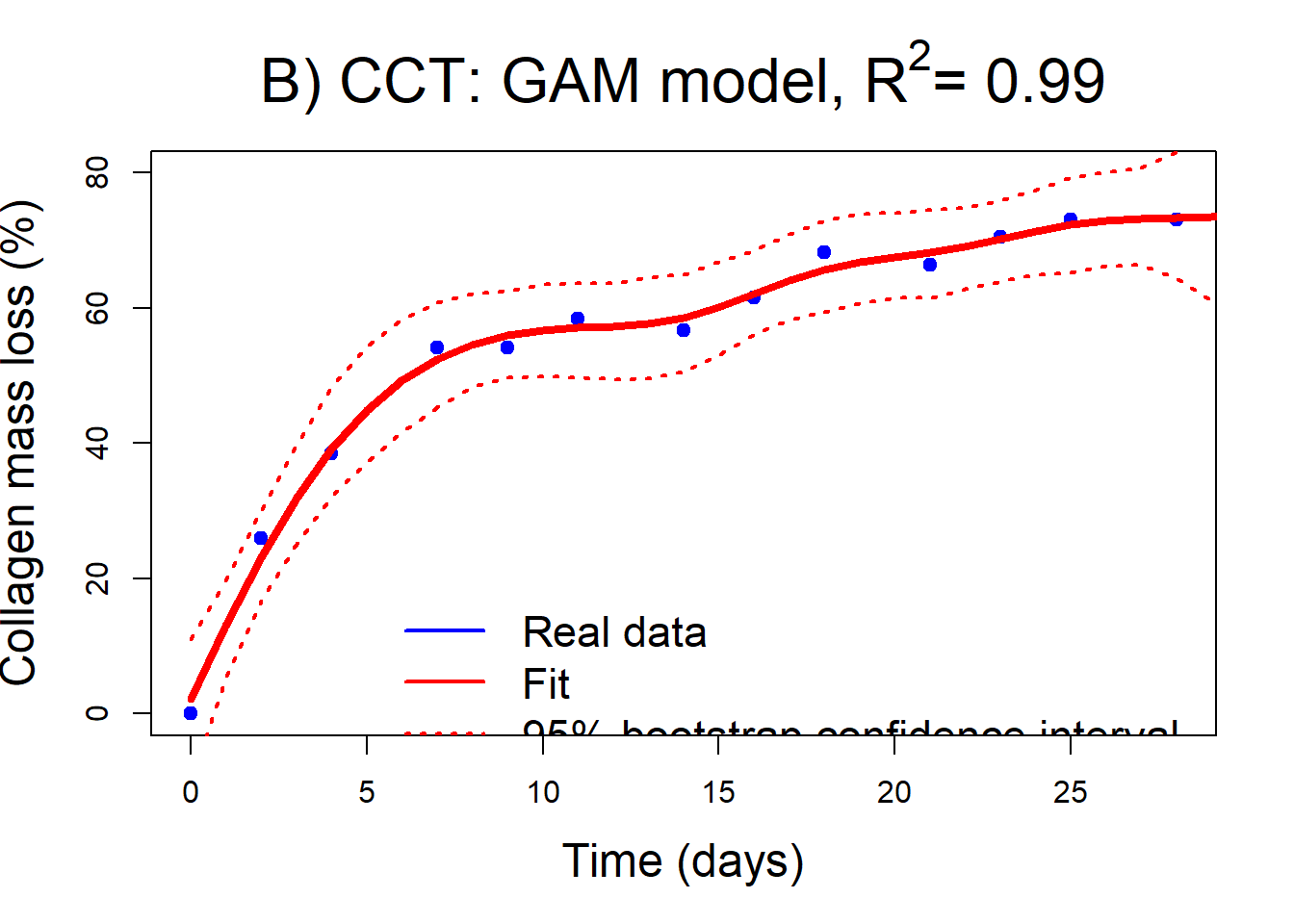

Supplement: Supplemental Information 1 — A html report is developed with R markdown and included with the corresponding files. Code and outputs are included. [file peerj-07-7233-s004.zip › Scripts_and_Outputs_files/figure-html/unnamed-chunk-7-1.png]

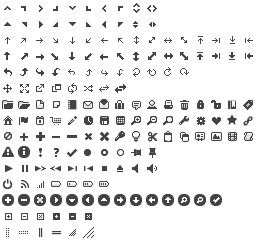

Supplement: Supplemental Information 1 — A html report is developed with R markdown and included with the corresponding files. Code and outputs are included. [file peerj-07-7233-s004.zip › Scripts_and_Outputs_files/jqueryui-1.11.4/images/ui-icons_444444_256x240.png]

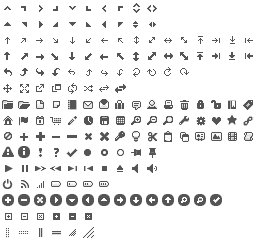

Supplement: Supplemental Information 1 — A html report is developed with R markdown and included with the corresponding files. Code and outputs are included. [file peerj-07-7233-s004.zip › Scripts_and_Outputs_files/jqueryui-1.11.4/images/ui-icons_555555_256x240.png]

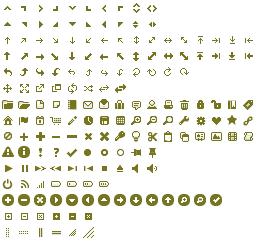

Supplement: Supplemental Information 1 — A html report is developed with R markdown and included with the corresponding files. Code and outputs are included. [file peerj-07-7233-s004.zip › Scripts_and_Outputs_files/jqueryui-1.11.4/images/ui-icons_777620_256x240.png]

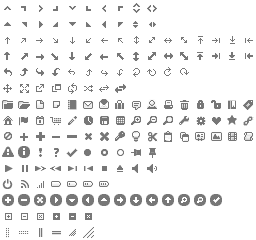

Supplement: Supplemental Information 1 — A html report is developed with R markdown and included with the corresponding files. Code and outputs are included. [file peerj-07-7233-s004.zip › Scripts_and_Outputs_files/jqueryui-1.11.4/images/ui-icons_777777_256x240.png]

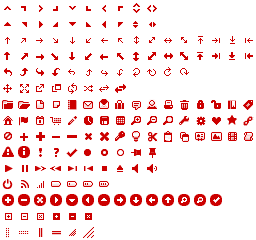

Supplement: Supplemental Information 1 — A html report is developed with R markdown and included with the corresponding files. Code and outputs are included. [file peerj-07-7233-s004.zip › Scripts_and_Outputs_files/jqueryui-1.11.4/images/ui-icons_cc0000_256x240.png]

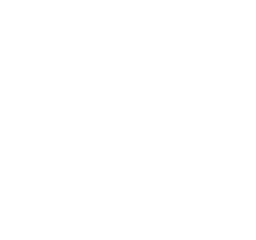

Supplement: Supplemental Information 1 — A html report is developed with R markdown and included with the corresponding files. Code and outputs are included. [file peerj-07-7233-s004.zip › Scripts_and_Outputs_files/jqueryui-1.11.4/images/ui-icons_ffffff_256x240.png]
