# Supplementary material for: An artificial-vision- and statistical-learning-based method for studying the biodegradation of type I collagen scaffolds in bone regeneration systems
Source: PeerJ. 2019 Jul 5;7:e7233. doi: 10.7717/peerj.7233 (PMC6613533; doi:10.7717/peerj.7233)
Supplement: Supplemental Information 1 — A html report is developed with R markdown and included with the corresponding files. Code and outputs are included. [file peerj-07-7233-s004.zip › Scripts_and_Outputs.html]

Artificial vision and statistical learning based method to study the biodegradation of type I collagen scaffolds in bone regeneration systems


# Artificial vision and statistical learning based method to study the biodegradation of type I collagen scaffolds in bone regeneration systems

### ***R scripts***

#### *Javier Tarrío Saavedra (javier.tarrio@udc.es)*

Universidade da Coruña  

#### *20 Agosto 2018*

### *Summary*

In this report, the scripts in R language corresponding to the manuscript entitled ``Artificial vision and statistical learning based method to study the biodegradation of type I collagen scaffolds in bone regeneration systems’’ are presented and commented.

## Descriptive analysis

```
load("D:/DisCoDuroPortatil/Yaroslava2/Yaroslava2/210218.RData")
```

### Study of the dependence between variables: Scatterplots

```
library(RcmdrMisc)
```

```
## Loading required package: car
```

```
## Loading required package: carData
```

```
## Loading required package: sandwich
```

```
scatterplotMatrix(~Area+Circ.+Mode+pesofresco+Time|Grupo,   
  regLine=FALSE, 
  smooth=FALSE, 
  diagonal=list(method="density"), 
  by.groups=TRUE, data=Dataset2[(Dataset2$Grupo=="CCO" |  
  Dataset2$Grupo=="CCT"),])
```

```
scatterplotMatrix(~Area+Circ.+Mode+pesofresco+Time| Grupo, 
  regLine=FALSE, 
  smooth=FALSE, 
  diagonal=list(method="density"), 
  by.groups=TRUE, data=Dataset2)
```

## Modeling of collagen mass loss vs time

### Nonparametric regression modeling (GAM)

A nonparametric regression model is applied to estimate the loss of collagen mass as a function of time, that is, to model the degree of degradation of the biomaterial. In particular, a base of tin plate splines is adjusted using the R mgcv library, designed for the estimation of generalized additive models (GAM). Confidence bands obtained by boot-trace resampling are included.

#### CCO dataset

```
library(mgcv)

gam0=gam(pesofresco2~s(Time),data=COO)
summary(gam0)
```

```
## 
## Family: gaussian 
## Link function: identity 
## 
## Formula:
## pesofresco2 ~ s(Time)
## 
## Parametric coefficients:
##             Estimate Std. Error t value Pr(>|t|)    
## (Intercept)   54.273      1.605   33.82 3.52e-09 ***
## ---
## Signif. codes:  0 '***' 0.001 '**' 0.01 '*' 0.05 '.' 0.1 ' ' 1
## 
## Approximate significance of smooth terms:
##           edf Ref.df     F  p-value    
## s(Time) 4.823   5.88 25.78 2.11e-06 ***
## ---
## Signif. codes:  0 '***' 0.001 '**' 0.01 '*' 0.05 '.' 0.1 ' ' 1
## 
## R-sq.(adj) =  0.926   Deviance explained = 95.6%
## GCV = 60.635  Scale est. = 33.477    n = 13
```

It is observed that the soft effect (none parametric relationship is assumed) of time on the mass loss of collagen is significant, explaining 93% of its total variability.

```
#  BOOTSTRAP CONFIDENCE INTERVALS

muhat<-predict.gam(gam0,type="response")

muhatg=muhat  # Pilot estimation
error=COO$pesofresco2-muhatg
error=error-mean(error)
sigma=sd(error) #estimates of \sigma

xgrid=seq(0,30,by=1)  # grid of points where I want to predict
pgrid <- predict.gam(gam0,data.frame(Time=xgrid),type="response")

x=COO$Time
##  BOOTSTRAP RESAMPLINGS
B=1000  # nº of resamplings
pgridb <- NULL
n=length(COO$Time)

for (iboot in 1:B)  {
  
  yboot=muhatg+rnorm(n)*sigma
  model=gam(yboot~s(x))
  pgridb<-
    
    cbind(pgridb,predict.gam(model,data.frame(x=xgrid),type="response"))
  
  
}


## QUANTILES

ngrid=length(xgrid)
qs<-rep(0,ngrid)
qi<-rep(0,ngrid)

for (i in 1:ngrid)
{
  qs[i]=quantile(pgridb[i,],probs=c(0.975))
  qi[i]=quantile(pgridb[i,],probs=c(0.025))
}


## CONFIDENCE INTERVALS
li=2*pgrid-qs
ls=2*pgrid-qi

## GRAPHICAL OUTPUT
a=predict.gam(gam0,type="response",newdata=data.frame(Time=seq(0,30)),se=TRUE)
plot(COO$Time,COO$pesofresco2,pch=19,col=4,
     ylim=c(0,80),xlab="Time (days)",cex.main=2,
     ylab="Collagen mass loss (%)",cex.lab=1.5,
     main=expression(paste("A) COO: GAM model, ",R^2,"= 0.93")))
lines(seq(0,30),a$fit,col=2,lwd=4)
lines(xgrid,li,col=2,lwd=2,lty=3)
lines(xgrid,ls,col=2,lwd=2,lty=3)
legend(5,20,c("Real data","Fit","95% bootstrap confidence interval"),
       bty="n",lty=c(1
                     ,1,3),lwd=2,col=c(4,2,2),cex=1.4)
```

#### CCT dataset

```
library(mgcv)

gam0=gam(pesofresco2~s(Time),data=CCT)
summary(gam0)
```

```
## 
## Family: gaussian 
## Link function: identity 
## 
## Formula:
## pesofresco2 ~ s(Time)
## 
## Parametric coefficients:
##             Estimate Std. Error t value Pr(>|t|)    
## (Intercept)   53.886      0.686   78.55 4.84e-10 ***
## ---
## Signif. codes:  0 '***' 0.001 '**' 0.01 '*' 0.05 '.' 0.1 ' ' 1
## 
## Approximate significance of smooth terms:
##           edf Ref.df     F p-value    
## s(Time) 6.171  7.306 118.8  <2e-16 ***
## ---
## Signif. codes:  0 '***' 0.001 '**' 0.01 '*' 0.05 '.' 0.1 ' ' 1
## 
## R-sq.(adj) =  0.986   Deviance explained = 99.3%
## GCV = 13.646  Scale est. = 6.1184    n = 13
```

```
#  BOOTSTRAP CONFIDENCE INTERVALS

muhat<-predict.gam(gam0,type="response")

muhatg=muhat  # Pilot estimation
error=COO$pesofresco2-muhatg
error=error-mean(error)
sigma=sd(error) #estimates of \sigma

xgrid=seq(0,30,by=1)  # grid of points where I want to predict
pgrid <- predict.gam(gam0,data.frame(Time=xgrid),type="response")

x=COO$Time
##  BOOTSTRAP RESAMPLINGS
B=1000  # nº of resamplings
pgridb <- NULL
n=length(COO$Time)

for (iboot in 1:B)  {
  
  yboot=muhatg+rnorm(n)*sigma
  model=gam(yboot~s(x))
  pgridb<-
    
    cbind(pgridb,predict.gam(model,data.frame(x=xgrid),type="response"))
  
  
}


## QUANTILES

ngrid=length(xgrid)
qs<-rep(0,ngrid)
qi<-rep(0,ngrid)

for (i in 1:ngrid)
{
  qs[i]=quantile(pgridb[i,],probs=c(0.975))
  qi[i]=quantile(pgridb[i,],probs=c(0.025))
}


## CONFIDENCE INTERVALS
li=2*pgrid-qs
ls=2*pgrid-qi

## GRAPHICAL OUTPUT
a=predict.gam(gam0,type="response",newdata=data.frame(Time=seq(0,30)),se=TRUE)
plot(CCT$Time,CCT$pesofresco2,pch=19,col=4,
     ylim=c(0,80),xlab="Time (days)",cex.main=2,
     ylab="Collagen mass loss (%)",cex.lab=1.5,
     main=expression(paste("B) CCT: GAM model, ",R^2,"= 0.99")))
lines(seq(0,30),a$fit,col=2,lwd=4)
lines(xgrid,li,col=2,lwd=2,lty=3)
lines(xgrid,ls,col=2,lwd=2,lty=3)
legend(5,20,c("Real data","Fit","95% bootstrap confidence interval"),
       bty="n",lty=c(1
                     ,1,3),lwd=2,col=c(4,2,2),cex=1.4)
```

#### Remarks

It is observed that the relationship between the lost mass and time is non-linear of sigmoid type. It can even be observed that the underlying model that relates to the loss of mass with time can be composed of a mixture of two non-linear functions, each one possibly related to a different degradation process. One of the alternatives for separating overlapping degradation processes is the adjustment of non-linear regression models based on the sum of logistic functions, widely used in thermogravimetry. We propose, therefore, the adjustment of a model composed of the mixture of two logistic functions to model the degradation of type I collagen when it is subject to degradation by growth and cell differentiation to osteocytes, from stem cells.

### Parametric regression modeling: Logistic mixture model

A logistics mixture model is adjusted by the following procedure:

1. The objective function to be minimized is defined according to the parameters that are to be estimated.
2. An initial solution is obtained from the adjustment of an evolutionary algorithm (R DEoptim package).
3. The non-linear regression model is adjusted based on the sum of two logistic from the nls2 package, obtaining an estimation of the parameters and their study of significance.
4. A dispersion diagram is obtained with the real data, the adjustment, its estimation and prediction intervals, also including the expression of the model with its coefficient of determination and the representation of the two logistic functions of which it is composed.

#### Fitting from the CCO dataset

The objective function to be minimized is defined according to the parameters that are to be estimated.

```
COO2=COO
MeanSquares1=function(param,X,Y){
  sum((COO2$pesofresco2-
         ((param[1]/(1+exp((param[2]-COO2$Time)/param[3])))+
            (param[4]/(1+exp((param[5]-COO2$Time)/param[6])))))^2)
}
```

An initial solution is obtained from the adjustment of an evolutionary algorithm (package R DEoptim).

```
library(DEoptim)
set.seed(1)
Optimizacion <- DEoptim(fn = MeanSquares1,
                        lower = c(0, -50, 1,0,-50,1),
                        upper = c(100,500,5,100, 500, 5),
                        control = list(itermax = 3000, NP = 200,trace=FALSE))

summary(Optimizacion)
```

```
## 
## ***** summary of DEoptim object ***** 
## best member   :  23.46286 16.26105 1.32327 50.2268 1.73404 1 
## best value    :  274.4669 
## after         :  3000 generations 
## fn evaluated  :  6002 times 
## *************************************
```

The non-linear regression model is adjusted based on the sum of two logistic from the nls2 package, obtaining an estimation of the parameters and their study of significance.

```
library("nlstools");library(nls2)
formulaExp <- as.formula(pesofresco2 ~ (Asym/(1+exp((xmid-Time)/scal))+
                                                Asym2/(1+exp((xmid2-Time)/scal2))))

preview(formulaExp, data = COO2,
            start = list(Asym =Optimizacion$optim$bestmem[[1]], 
                          xmid = Optimizacion$optim$bestmem[[2]],
                          scal = Optimizacion$optim$bestmem[[3]],
                          Asym2 =Optimizacion$optim$bestmem[[4]], 
                          xmid2 = Optimizacion$optim$bestmem[[5]],
                          scal2 = Optimizacion$optim$bestmem[[6]]))
```

```
## 
## RSS:  274
```

```
fm1 <- nls2(formulaExp, 
             data = COO2,control = nls.control(maxiter = 1024),
             algorithm = "brute-force",
             start = list(Asym =Optimizacion$optim$bestmem[[1]], 
                       xmid = Optimizacion$optim$bestmem[[2]],
                       scal = Optimizacion$optim$bestmem[[3]],
                       Asym2 =Optimizacion$optim$bestmem[[4]], 
                       xmid2 = Optimizacion$optim$bestmem[[5]],
                       scal2 = Optimizacion$optim$bestmem[[6]]))

for(i in 1:1000){
  fm1 <- nls2(formulaExp, 
            data = COO2,control = nls.control(maxiter = 1024),
            algorithm = "random-search",
            start = summary(fm1)$parameters[,1])                         
}

 overview(fm1)
```

```
## 
## ------
## Formula: pesofresco2 ~ (Asym/(1 + exp((xmid - Time)/scal)) + Asym2/(1 + 
##     exp((xmid2 - Time)/scal2)))
## 
## Parameters:
##       Estimate Std. Error t value Pr(>|t|)    
## Asym   23.4629     5.4631   4.295  0.00359 ** 
## xmid   16.2610     1.3286  12.239 5.57e-06 ***
## scal    1.3233     1.2447   1.063  0.32304    
## Asym2  50.2268     3.8997  12.880 3.95e-06 ***
## xmid2   1.7340     0.4812   3.604  0.00870 ** 
## scal2   1.0000     0.4464   2.240  0.06006 .  
## ---
## Signif. codes:  0 '***' 0.001 '**' 0.01 '*' 0.05 '.' 0.1 ' ' 1
## 
## Residual standard error: 6.262 on 7 degrees of freedom
## 
## Number of iterations to convergence: 6 
## Achieved convergence tolerance: NA
## 
## ------
## Residual sum of squares: 274 
## 
## ------
## t-based confidence interval:
##              2.5%     97.5%
## Asym  10.54463750 36.381082
## xmid  13.11939687 19.402701
## scal  -1.62008502  4.266634
## Asym2 41.00555015 59.448048
## xmid2  0.59623936  2.871837
## scal2 -0.05551794  2.055518
## 
## ------
## Correlation matrix:
##              Asym        xmid        scal      Asym2      xmid2      scal2
## Asym   1.00000000 -0.07048467  0.54938194 -0.7824615 -0.3475206 -0.2939884
## xmid  -0.07048467  1.00000000 -0.02002914  0.4154797  0.1848332  0.1567736
## scal   0.54938194 -0.02002914  1.00000000 -0.4293289 -0.1894080 -0.1573721
## Asym2 -0.78246148  0.41547969 -0.42932886  1.0000000  0.4445889  0.3770579
## xmid2 -0.34752059  0.18483321 -0.18940799  0.4445889  1.0000000  0.1258475
## scal2 -0.29398841  0.15677359 -0.15737213  0.3770579  0.1258475  1.0000000
```

Goodness of fit from calculing \(R^2\):

```
 R_2=(sum((CCT$pesofresco2-mean(COO$pesofresco2))
          *((50.23/(1+exp((1.73-COO$Time)/1.00))+23.46/(1+exp((16.26-COO$Time)/1.32)))-mean(COO$pesofresco2))))^2/
   (sum((COO$pesofresco2-mean(COO$pesofresco2))^2)*
      sum(((50.23/(1+exp((1.73-COO$Time)/1.00))+23.46/(1+exp((16.26-COO$Time)/1.32)))-mean(COO$pesofresco2))^2))
R_2
```

```
## [1] 0.9444592
```

A dispersion diagram is obtained with the real data, the adjustment, its estimation and prediction intervals, also including the expression of the model with its coefficient of determination and the representation of the two logistic functions of which it is composed.

```
# Gráfica con IC
windows(9,8)
library(RColorBrewer)
library(investr)
blues <- brewer.pal(9, "Blues") # Voy a elegir el azul
plotFit(fm1, interval = "both", pch = 19, shade = TRUE,lwd.fit = 2,
        col.fit = "red",col.conf = blues[4], col.pred = blues[2],
        xlab="Time (days)", ylab="Mass loss (%)",cex.lab=1.5,
        cex.axis=1.3,cex.main=1.3,
        main=expression(paste("\ny =", 50.23/(1+exp((1.73-x)/1.00))+23.46/(1+exp((16.26-x)/1.32)),"; ",
                              R^2,"= 0.94")))
lines(seq(0,28),(50.23/(1+exp((1.73-seq(0,28))/1.00))),col=9,lty=2,lwd=2)
lines(seq(0,28),(23.46/(1+exp((16.26-seq(0,28))/1.32))),col=6,lty=2,lwd=2)

legend(-1.5,97,bty="n", col=c(2,blues[4],blues[2],1,6),cex=1.3, lty=c(1,1,1,2,2),  lwd=2,
       c("Fit","Estimation confidence interval at 95%",
         "Prediction confidence interval at 95%",
         "First logistic component",
         "Second logistic component"),)
```

#### Fitting from the CCT dataset

The objective function to be minimized is defined according to the parameters that are to be estimated.

```
MeanSquares1=function(param,X,Y){
  sum((CCT$pesofresco2-
         ((param[1]/(1+exp((param[2]-CCT$Time)/param[3])))+
            (param[4]/(1+exp((param[5]-CCT$Time)/param[6])))))^2)
}
```

An initial solution is obtained from the adjustment of an evolutionary algorithm (package R DEoptim).

```
library(DEoptim)
Optimizacion <- DEoptim(fn = MeanSquares1,
                        lower = c(0, 0, 0,0,0,0),
                        upper = c(100, 1000, 10000,100, 1000, 10000),
                        control = list(itermax = 10500, NP = 120,trace=FALSE))

summary(Optimizacion)
```

```
## 
## ***** summary of DEoptim object ***** 
## best member   :  52.50187 2.47676 1.15464 21.65256 16.88102 3.65542 
## best value    :  98.97338 
## after         :  10500 generations 
## fn evaluated  :  21002 times 
## *************************************
```

The non-linear regression model is adjusted based on the sum of two logistic from the nls function, obtaining an estimation of the parameters and their study of significance.

```
library("nlstools")
formulaExp <- as.formula(pesofresco2 ~ (Asym/(1+exp((xmid-Time)/scal))+                     Asym2/(1+exp((xmid2-Time)/scal2))))

preview(formulaExp, data = CCT,
        start = list(Asym =Optimizacion$optim$bestmem[[1]], 
                     xmid = Optimizacion$optim$bestmem[[2]],
                     scal = Optimizacion$optim$bestmem[[3]],
                     Asym2 =Optimizacion$optim$bestmem[[4]], 
                     xmid2 = Optimizacion$optim$bestmem[[5]],
                     scal2 = Optimizacion$optim$bestmem[[6]]))
```

```
## 
## RSS:  99
```

```
fm1 <- nls(formulaExp, 
           data = CCT,control = nls.control(maxiter = 1024),
           start = list(Asym =Optimizacion$optim$bestmem[[1]], 
                        xmid = Optimizacion$optim$bestmem[[2]],
                        scal = Optimizacion$optim$bestmem[[3]],
                        Asym2 =Optimizacion$optim$bestmem[[4]], 
                        xmid2 = Optimizacion$optim$bestmem[[5]],
                        scal2 = Optimizacion$optim$bestmem[[6]]))
overview(fm1)
```

```
## 
## ------
## Formula: pesofresco2 ~ (Asym/(1 + exp((xmid - Time)/scal)) + Asym2/(1 + 
##     exp((xmid2 - Time)/scal2)))
## 
## Parameters:
##       Estimate Std. Error t value Pr(>|t|)    
## Asym   52.5019     7.6993   6.819 0.000249 ***
## xmid    2.4768     0.3700   6.694 0.000279 ***
## scal    1.1546     0.3366   3.430 0.010981 *  
## Asym2  21.6526    12.5209   1.729 0.127378    
## xmid2  16.8810     3.0399   5.553 0.000857 ***
## scal2   3.6554     3.7772   0.968 0.365400    
## ---
## Signif. codes:  0 '***' 0.001 '**' 0.01 '*' 0.05 '.' 0.1 ' ' 1
## 
## Residual standard error: 3.76 on 7 degrees of freedom
## 
## Number of iterations to convergence: 0 
## Achieved convergence tolerance: 1.901e-07
## 
## ------
## Residual sum of squares: 99 
## 
## ------
## t-based confidence interval:
##             2.5%     97.5%
## Asym  34.2958785 70.707858
## xmid   1.6018355  3.351686
## scal   0.3587025  1.950580
## Asym2 -7.9546908 51.259817
## xmid2  9.6927733 24.069266
## scal2 -5.2761710 12.587012
## 
## ------
## Correlation matrix:
##             Asym       xmid       scal      Asym2      xmid2      scal2
## Asym   1.0000000  0.6371874  0.6329383 -0.9276410  0.5541616 -0.8702963
## xmid   0.6371874  1.0000000  0.4644863 -0.5293525  0.4501796 -0.4349454
## scal   0.6329383  0.4644863  1.0000000 -0.5561191  0.3913552 -0.4855947
## Asym2 -0.9276410 -0.5293525 -0.5561191  1.0000000 -0.2436847  0.9504313
## xmid2  0.5541616  0.4501796  0.3913552 -0.2436847  1.0000000 -0.2058924
## scal2 -0.8702963 -0.4349454 -0.4855947  0.9504313 -0.2058924  1.0000000
```

Goodness of fit from calculin the \(R^2\):

```
plotfit(fm1,smooth=TRUE)
```

```
R_2=(sum((CCT$pesofresco2-mean(COO$pesofresco2))
         *((52.5019/(1+exp((2.4768-CCT$Time)/1.1546))+21.6526/(1+exp((16.8810-CCT$Time)/3.6554)))-mean(COO$pesofresco2))))^2/
  (sum((COO$pesofresco2-mean(COO$pesofresco2))^2)*
     sum(((52.5019/(1+exp((2.4768-CCT$Time)/1.1546))+21.6526/(1+exp((16.8810-CCT$Time)/3.6554)))-mean(COO$pesofresco2))^2))
R_2
```

```
## [1] 0.975124
```

A dispersion diagram is obtained with the real data, the adjustment, its estimation and prediction intervals, also including the expression of the model with its coefficient of determination and the representation of the two logistic functions of which it is composed.

```
# Gráfica con IC
windows(9,8)
library(RColorBrewer)
library(investr)

blues <- brewer.pal(9, "Blues") # Voy a elegir el azul
plotFit(fm1, interval = "both", pch = 19, shade = TRUE,lwd.fit = 2,
        col.fit = "red",col.conf = blues[4], col.pred = blues[2],
        xlab="Time (days)", ylab="Mass loss (%)",cex.lab=1.5,
        cex.axis=1.3,cex.main=1.3,
        main=expression(paste("\ny =", 52.5019/(1+exp((2.4768-x)/1.1546))+21.6526/(1+exp((16.8810-x)/3.6554)) ,
                              R^2,"= 0.98")))
lines(seq(0,28),(52.5019/(1+exp((2.4768-seq(0,28))/1.1546))),col=9,lty=2,lwd=2)
lines(seq(0,28),(21.6526/(1+exp((16.8810-seq(0,28))/3.6554))),col=6,lty=2,lwd=2)

legend(-2,97,bty="n", col=c(2,blues[4],blues[2],1,6),cex=1.3, lty=c(1,1,1,2,2),  lwd=2,
       c("Fit","Estimation confidence interval at 95%",
         "Prediction confidence interval at 95%",
         "First logistic component",
         "Second logistic component"))
```

Study of the parameters using the bootstrap method:

```
set.seed(4)
O2K.jack1 <- nlsBoot(fm1)
```

```
## Warning in nlsBoot(fm1): The fit did not converge 70 times during
## bootstrapping
```

```
summary(O2K.jack1)
```

```
## 
## ------
## Bootstrap statistics
##        Estimate Std. error
## Asym  50.843934  5.9644870
## xmid   2.529998  0.2650045
## scal   1.100230  0.2779857
## Asym2 27.025514 47.7170126
## xmid2 17.229974  4.2755710
## scal2  4.037186  2.6069552
## 
## ------
## Median of bootstrap estimates and percentile confidence intervals
##          Median       2.5%     97.5%
## Asym  52.796040 36.1313580 58.400565
## xmid   2.521091  2.0578047  3.079044
## scal   1.099805  0.5631438  1.631135
## Asym2 21.555539 13.5392577 55.246406
## xmid2 16.973953 10.7525587 26.855772
## scal2  3.347424  0.6866024 10.409565
```

## Relationship between micrographs extracted features and time

### Fittings corresponding to the CCO group

#### Area vs. time

The relationship can be modeled with a nonlinear regression model based on logistic fucntion.

```
MeanSquares1=function(param,X,Y){
  sum((COO$Area-(param[1]/(1+exp((param[2]-CO$Time)/param[3]))))^2)
}

library(DEoptim)
Optimizacion <- DEoptim(fn = MeanSquares1,
                        lower = c(0, 0, 0),
                        upper = c(10000, 1000, 10000),
                        control = list(itermax = 10500, NP = 120,trace=FALSE))

summary(Optimizacion)
```

```
## 
## ***** summary of DEoptim object ***** 
## best member   :  880.4108 9.25149 1.7961 
## best value    :  452294.7 
## after         :  10500 generations 
## fn evaluated  :  21002 times 
## *************************************
```

```
library(minpack.lm)
fm1 <- nlsLM(Area ~ Asym/(1+exp((xmid-Time)/scal)), 
             data = COO,control = nls.control(maxiter = 1024),
             start = c(Asym =Optimizacion$optim$bestmem[[1]], 
                       xmid = Optimizacion$optim$bestmem[[2]],
                       scal = Optimizacion$optim$bestmem[[3]]))


for(i in 1:10000){
  fm1DNaseCreep1 <- nlsLM(Area ~ Asym/(1+exp((xmid-Time)/scal)), 
                          data = COO,control = nls.control(maxiter = 1024),
                          start = summary(fm1)$parameters[,1])
}


summary(fm1)
```

```
## 
## Formula: Area ~ Asym/(1 + exp((xmid - Time)/scal))
## 
## Parameters:
##      Estimate Std. Error t value Pr(>|t|)    
## Asym  880.411     89.875   9.796 1.92e-06 ***
## xmid    9.251      1.281   7.224 2.85e-05 ***
## scal    1.796      1.221   1.471    0.172    
## ---
## Signif. codes:  0 '***' 0.001 '**' 0.01 '*' 0.05 '.' 0.1 ' ' 1
## 
## Residual standard error: 212.7 on 10 degrees of freedom
## 
## Number of iterations to convergence: 1 
## Achieved convergence tolerance: 1.49e-08
```

```
R_2=(sum((COO$Area-mean(COO$Area))
         *(880.411/(1+exp((9.251-COO$Time)/1.796))-mean(COO$Area))))^2/
  (sum((COO$Area-mean(COO$Area))^2)*
     sum((880.411/(1+exp((9.251-COO$Time)/1.796))-mean(COO$Area))^2))
R_2 #=0.7521112
```

```
## [1] 0.7521112
```

```
# Plot wit CI
library(RColorBrewer)
library(investr)
windows(9,8)
blues <- brewer.pal(9, "Blues") 
plotFit(fm1, interval = "both", pch = 19, shade = TRUE,lwd.fit = 2,
        col.fit = "red",col.conf = blues[4], col.pred = blues[2],
        xlab="Time (days)", ylab=expression(paste("Area (",mu,m^2,")")),
        cex.lab=1.1,
        cex.axis=1.1,
        main=expression(paste("\ny =", 880.411/(1+exp((9.251-x)/1.796)),"; ",
                              R^2,"= 0.75")))
legend(12,30,bty="n", col=c(2,blues[4],blues[2]),cex=1.2, lty=c(1,1,1),  lwd=2,
       c("Fit","Estimation confidence interval at 95%","Prediction confidence interval at 95%"),)
```

#### Circularity vs. time

```
COO$pesofresco3=log(COO$pesofresco2)
COO$Circ.1=log(COO$Circ.)
lm=lm(Circ.1~(Time), data=COO)
outlierTest(lm)
```

```
## No Studentized residuals with Bonferonni p < 0.05
## Largest |rstudent|:
##   rstudent unadjusted p-value Bonferonni p
## 4 3.351572          0.0073459     0.095496
```

```
lm=lm(Circ.1~(Time), data=COO[-c(4),]);summary(lm)
```

```
## 
## Call:
## lm(formula = Circ.1 ~ (Time), data = COO[-c(4), ])
## 
## Residuals:
##      Min       1Q   Median       3Q      Max 
## -0.94890 -0.38648 -0.04451  0.25743  1.08470 
## 
## Coefficients:
##             Estimate Std. Error t value Pr(>|t|)    
## (Intercept) -3.03196    0.36174  -8.382 7.81e-06 ***
## Time        -0.13562    0.02156  -6.289 9.03e-05 ***
## ---
## Signif. codes:  0 '***' 0.001 '**' 0.01 '*' 0.05 '.' 0.1 ' ' 1
## 
## Residual standard error: 0.6612 on 10 degrees of freedom
## Multiple R-squared:  0.7982, Adjusted R-squared:  0.778 
## F-statistic: 39.56 on 1 and 10 DF,  p-value: 9.032e-05
```

```
# Scatterplot with CI
library(RColorBrewer)
library(investr)
windows(9,8)
blues <- brewer.pal(9, "Blues") 
plotFit(lm, interval = "both", pch = 19, shade = TRUE,lwd.fit = 2,
        col.fit = "red",col.conf = blues[4], col.pred = blues[2],
        ylab="ln(Circularity)", 
        xlab="Time (days)",cex.lab=1.3,
        cex.axis=1.3,
        main=expression(paste("\nln(y) =", -3.0320-0.1356*x,"; ",
                              R^2,"= 0.80")))
```

```
## Warning in predFit.lm(object, newdata = xgrid, interval = "prediction", :
## predictions on current data refer to _future_ responses
```

```
legend(0,-7,bty="n", col=c(2,blues[4],blues[2]),cex=1.2, lty=c(1,1,1),  lwd=2,
       c("Fit","Estimation confidence interval at 95%","Prediction confidence interval at 95%"),)
```

### Fittings corresponding to the CCT group

#### Area vs. time

```
MeanSquares1=function(param,X,Y){
  sum((CCT$Area-(param[1]/(1+exp((param[2]-CO$Time)/param[3]))))^2)
}

library(DEoptim)
Optimizacion <- DEoptim(fn = MeanSquares1,
                        lower = c(0, 0, 0),
                        upper = c(10000, 1000, 10000),
                        control = list(itermax = 10500, NP = 120,trace=FALSE))

summary(Optimizacion)
```

```
## 
## ***** summary of DEoptim object ***** 
## best member   :  1874.284 4.19752 5.74517 
## best value    :  12805043 
## after         :  10500 generations 
## fn evaluated  :  21002 times 
## *************************************
```

```
library(minpack.lm)
fm1 <- nlsLM(Area ~ Asym/(1+exp((xmid-Time)/scal)), 
             data = CCT,control = nls.control(maxiter = 1024),
             start = c(Asym =Optimizacion$optim$bestmem[[1]], 
                       xmid = Optimizacion$optim$bestmem[[2]],
                       scal = Optimizacion$optim$bestmem[[3]]))


for(i in 1:10000){
  fm1DNaseCreep1 <- nlsLM(Area ~ Asym/(1+exp((xmid-Time)/scal)), 
                          data = CCT,control = nls.control(maxiter = 1024),
                          start = summary(fm1)$parameters[,1])
}

summary(fm1)
```

```
## 
## Formula: Area ~ Asym/(1 + exp((xmid - Time)/scal))
## 
## Parameters:
##      Estimate Std. Error t value Pr(>|t|)  
## Asym 1874.284    832.526   2.251   0.0481 *
## xmid    4.198      8.467   0.496   0.6308  
## scal    5.745     10.674   0.538   0.6022  
## ---
## Signif. codes:  0 '***' 0.001 '**' 0.01 '*' 0.05 '.' 0.1 ' ' 1
## 
## Residual standard error: 1132 on 10 degrees of freedom
## 
## Number of iterations to convergence: 1 
## Achieved convergence tolerance: 1.49e-08
```

```
R_2=(sum((CCT$Area-mean(CCT$Area))
         *(1874.284/(1+exp((4.198-CCT$Time)/5.745))-mean(CCT$Area))))^2/
  (sum((CCT$Area-mean(CCT$Area))^2)*
     sum((1874.284/(1+exp((4.198-CCT$Time)/5.745))-mean(CCT$Area))^2))
R_2 #=0.1476217
```

```
## [1] 0.1476217
```

```
# Plot with CI
library(RColorBrewer)
library(investr)
windows(9,8)
blues <- brewer.pal(9, "Blues") # Voy a elegir el azul
plotFit(fm1, interval = "both", pch = 19, shade = TRUE,lwd.fit = 2,
        col.fit = "red",col.conf = blues[4], col.pred = blues[2],
        xlab="Time (days)", ylab=expression(paste("Area (",mu,m^2,")")),cex.lab=1.1,
        cex.axis=1.1,
        main=expression(paste("\ny =", 1874.284/(1+exp((4.198-x)/5.745)),"; ",
                              R^2,"= 0.15")))
legend(10,-1000,bty="n", col=c(2,blues[4],blues[2]),cex=1.2, lty=c(1,1,1),  lwd=2,
       c("Fit","Estimation confidence interval at 95%","Prediction confidence interval at 95%"),)
```

#### Circularity vs. time

```
CCT$pesofresco3=log(CCT$pesofresco2)
CCT$Circ.1=log(CCT$Circ.)
CCT$pesofresco4=CCT$pesofresco3
CCT$pesofresco4[1]=-41.4

lm=lm(Circ.1~(Time), data=CCT);summary(lm)
```

```
## 
## Call:
## lm(formula = Circ.1 ~ (Time), data = CCT)
## 
## Residuals:
##     Min      1Q  Median      3Q     Max 
## -0.6842 -0.6062 -0.2877  0.4341  1.4269 
## 
## Coefficients:
##             Estimate Std. Error t value Pr(>|t|)    
## (Intercept) -4.71297    0.36730 -12.831 5.83e-08 ***
## Time        -0.05444    0.02263  -2.406   0.0349 *  
## ---
## Signif. codes:  0 '***' 0.001 '**' 0.01 '*' 0.05 '.' 0.1 ' ' 1
## 
## Residual standard error: 0.7115 on 11 degrees of freedom
## Multiple R-squared:  0.3448, Adjusted R-squared:  0.2853 
## F-statistic: 5.789 on 1 and 11 DF,  p-value: 0.03486
```

```
# # Gráfica con IC
library(RColorBrewer)
library(investr)
windows(9,8)
blues <- brewer.pal(9, "Blues") 
plotFit(lm, interval = "both", pch = 19, shade = TRUE,lwd.fit = 2,
        col.fit = "red",col.conf = blues[4], col.pred = blues[2],
        ylab="ln(Circularity)", 
        xlab="Time (days)",cex.lab=1.3,
        cex.axis=1.3,
        main=expression(paste("\nln(y) =", -4.713-0.0544*x,"; ",
                              R^2,"= 0.34")))
```

```
## Warning in predFit.lm(object, newdata = xgrid, interval = "prediction", :
## predictions on current data refer to _future_ responses
```

```
legend(0,-7,bty="n", col=c(2,blues[4],blues[2]),cex=1.2, lty=c(1,1,1),  lwd=2,
       c("Fit","Estimation confidence interval at 95%","Prediction confidence interval at 95%"),)
```

## Modeling of collagen mass loss as a function of micrographs area or circularity

### Fittings corresponding to CCO group

#### Mass loss vs. area

```
COO2=COO[-1,] # Observations for which the logarithms are not defined are removed from dataset.
COO2$Area2=log(COO2$Area)
lm=lm(pesofresco2~Area2, data=COO2);summary(lm)
```

```
## 
## Call:
## lm(formula = pesofresco2 ~ Area2, data = COO2)
## 
## Residuals:
##      Min       1Q   Median       3Q      Max 
## -12.7569  -3.6767   0.7647   3.1427  14.6564 
## 
## Coefficients:
##             Estimate Std. Error t value Pr(>|t|)    
## (Intercept)   -5.217     13.547  -0.385 0.708233    
## Area2         10.578      2.204   4.800 0.000724 ***
## ---
## Signif. codes:  0 '***' 0.001 '**' 0.01 '*' 0.05 '.' 0.1 ' ' 1
## 
## Residual standard error: 8.234 on 10 degrees of freedom
## Multiple R-squared:  0.6973, Adjusted R-squared:  0.667 
## F-statistic: 23.04 on 1 and 10 DF,  p-value: 0.0007239
```

```
# Scatterplot with CI
library(RColorBrewer)
library(investr)
windows(9,8)
blues <- brewer.pal(9, "Blues") # Voy a elegir el azul
plotFit(lm, interval = "both", pch = 19, shade = TRUE,lwd.fit = 2,
        col.fit = "red",col.conf = blues[4], col.pred = blues[2],
        xlab="ln(Area)", ylab="Mass loss (%)",cex.lab=1.3,
        cex.axis=1.3,
        main=expression(paste("\ny =",-5.217+10.578,"; ", R^2,"= 0.70")))
```

```
## Warning in predFit.lm(object, newdata = xgrid, interval = "prediction", :
## predictions on current data refer to _future_ responses
```

```
        legend(5,30,bty="n", col=c(2,blues[4],blues[2]),cex=1.2, lty=c(1,1,1),  lwd=2,
               c("Fit","Estimation confidence interval at 95%","Prediction confidence interval at 95%"),)
```

#### Mass loss vs. circularity

```
COO$pesofresco3=log(COO$pesofresco2)
        COO$Circ.1=log(COO$Circ.)
        lm=lm(pesofresco2~(Circ.1), data=COO);summary(lm)
```

```
## 
## Call:
## lm(formula = pesofresco2 ~ (Circ.1), data = COO)
## 
## Residuals:
##     Min      1Q  Median      3Q     Max 
## -26.801  -5.643  -2.380   6.229  29.630 
## 
## Coefficients:
##             Estimate Std. Error t value Pr(>|t|)   
## (Intercept)    5.853     11.950   0.490  0.63391   
## Circ.1       -10.287      2.409  -4.271  0.00132 **
## ---
## Signif. codes:  0 '***' 0.001 '**' 0.01 '*' 0.05 '.' 0.1 ' ' 1
## 
## Residual standard error: 13.63 on 11 degrees of freedom
## Multiple R-squared:  0.6238, Adjusted R-squared:  0.5896 
## F-statistic: 18.24 on 1 and 11 DF,  p-value: 0.001319
```

```
        outlierTest(lm)
```

```
##   rstudent unadjusted p-value Bonferonni p
## 4 4.695403         0.00084746     0.011017
```

```
        lm=lm(pesofresco2~(Circ.1), data=COO[-c(4),]);summary(lm)
```

```
## 
## Call:
## lm(formula = pesofresco2 ~ (Circ.1), data = COO[-c(4), ])
## 
## Residuals:
##     Min      1Q  Median      3Q     Max 
## -11.636  -5.813   1.540   5.179  11.570 
## 
## Coefficients:
##             Estimate Std. Error t value Pr(>|t|)    
## (Intercept)  -19.352      8.822  -2.193    0.053 .  
## Circ.1       -14.866      1.715  -8.667  5.8e-06 ***
## ---
## Signif. codes:  0 '***' 0.001 '**' 0.01 '*' 0.05 '.' 0.1 ' ' 1
## 
## Residual standard error: 7.984 on 10 degrees of freedom
## Multiple R-squared:  0.8825, Adjusted R-squared:  0.8708 
## F-statistic: 75.11 on 1 and 10 DF,  p-value: 5.803e-06
```

```
        # Gráfica con IC
        library(RColorBrewer)
        library(investr)
        windows(9,8)
        blues <- brewer.pal(9, "Blues") # Voy a elegir el azul
        plotFit(lm, interval = "both", pch = 19, shade = TRUE,lwd.fit = 2,
                col.fit = "red",col.conf = blues[4], col.pred = blues[2],
                xlab="ln(Circularity)", 
                ylab="Mass loss (%)",cex.lab=1.3,
                cex.axis=1.3,
                main=expression(paste("\ny =", -19.35-14.87*ln(x),"; ",
                                      R^2,"= 0.88")))
```

```
## Warning in predFit.lm(object, newdata = xgrid, interval = "prediction", :
## predictions on current data refer to _future_ responses
```

```
        legend(-6.8,3.6,bty="n", col=c(2,blues[4],blues[2]),cex=1.2, lty=c(1,1,1),  lwd=2,
               c("Fit","Estimation confidence interval at 95%","Prediction confidence interval at 95%"),)
```

### Fittings corresponding to CCT group

#### Mass loss vs. area

```
CCT$Area2=log(CCT$Area)
lm=lm(pesofresco2~Area2, data=CCT);summary(lm)
```

```
## 
## Call:
## lm(formula = pesofresco2 ~ Area2, data = CCT)
## 
## Residuals:
##     Min      1Q  Median      3Q     Max 
## -32.597  -7.029   1.085   8.063  32.445 
## 
## Coefficients:
##             Estimate Std. Error t value Pr(>|t|)  
## (Intercept)  -54.458     53.586  -1.016   0.3313  
## Area2         15.439      7.599   2.032   0.0671 .
## ---
## Signif. codes:  0 '***' 0.001 '**' 0.01 '*' 0.05 '.' 0.1 ' ' 1
## 
## Residual standard error: 18.87 on 11 degrees of freedom
## Multiple R-squared:  0.2728, Adjusted R-squared:  0.2067 
## F-statistic: 4.127 on 1 and 11 DF,  p-value: 0.06707
```

```
# 
# Scatterplot with CI
library(RColorBrewer)
library(investr)
blues <- brewer.pal(9, "Blues") # Voy a elegir el azul
plotFit(lm, interval = "both", pch = 19, shade = TRUE,lwd.fit = 2,
        col.fit = "red",col.conf = blues[4], col.pred = blues[2],
        xlab="ln(Area)", ylab="Mass loss (%)",cex.lab=1.3,
        cex.axis=1.3,
        main=expression(paste("\ny =",-54.458+15.439,"; ",
                              R^2,"= 0.27")))
```

```
## Warning in predFit.lm(object, newdata = xgrid, interval = "prediction", :
## predictions on current data refer to _future_ responses
```

```
legend(6.5,5,bty="n", col=c(2,blues[4],blues[2]),cex=1.2, lty=c(1,1,1),  lwd=2,
       c("Fit","Estimation confidence interval at 95%","Prediction confidence interval at 95%"))
```

#### Mass loss vs. circularity

```
CCT$pesofresco3=log(CCT$pesofresco2)
        CCT$Circ.1=log(CCT$Circ.)
        CCT$pesofresco4=CCT$pesofresco3
        CCT$pesofresco4[1]=-41.4
        lm=lm(pesofresco2~(Circ.1), data=CCT);summary(lm)
```

```
## 
## Call:
## lm(formula = pesofresco2 ~ (Circ.1), data = CCT)
## 
## Residuals:
##     Min      1Q  Median      3Q     Max 
## -21.698  -6.935  -4.413  10.633  21.449 
## 
## Coefficients:
##             Estimate Std. Error t value Pr(>|t|)    
## (Intercept)  -57.406     24.576  -2.336 0.039457 *  
## Circ.1       -20.389      4.454  -4.578 0.000793 ***
## ---
## Signif. codes:  0 '***' 0.001 '**' 0.01 '*' 0.05 '.' 0.1 ' ' 1
## 
## Residual standard error: 12.98 on 11 degrees of freedom
## Multiple R-squared:  0.6558, Adjusted R-squared:  0.6245 
## F-statistic: 20.96 on 1 and 11 DF,  p-value: 0.000793
```

```
        outlierTest(lm)
```

```
## No Studentized residuals with Bonferonni p < 0.05
## Largest |rstudent|:
##     rstudent unadjusted p-value Bonferonni p
## 22 -1.964424           0.077862           NA
```

```
        lm=lm(pesofresco2~(Circ.1), data=CCT[-2,]);summary(lm)
```

```
## 
## Call:
## lm(formula = pesofresco2 ~ (Circ.1), data = CCT[-2, ])
## 
## Residuals:
##     Min      1Q  Median      3Q     Max 
## -13.301  -7.316  -4.960   9.835  19.240 
## 
## Coefficients:
##             Estimate Std. Error t value Pr(>|t|)    
## (Intercept)  -50.861     22.147  -2.297 0.044514 *  
## Circ.1       -19.525      3.992  -4.891 0.000632 ***
## ---
## Signif. codes:  0 '***' 0.001 '**' 0.01 '*' 0.05 '.' 0.1 ' ' 1
## 
## Residual standard error: 11.57 on 10 degrees of freedom
## Multiple R-squared:  0.7052, Adjusted R-squared:  0.6757 
## F-statistic: 23.92 on 1 and 10 DF,  p-value: 0.0006316
```

```
        # Gráfica con IC
        library(RColorBrewer)
        library(investr)
        windows(9,8)
        blues <- brewer.pal(9, "Blues") # Voy a elegir el azul
        plotFit(lm, interval = "both", pch = 19, shade = TRUE,lwd.fit = 2,
                col.fit = "red",col.conf = blues[4], col.pred = blues[2],
                xlab="ln(Circularity)", 
                ylab="Mass loss (%)",cex.lab=1.3,
                cex.axis=1.3,
                main=expression(paste("\ny =", -50.86-19.23*ln(x),"; ",
                                      R^2,"= 0.71")))
```

```
## Warning in predFit.lm(object, newdata = xgrid, interval = "prediction", :
## predictions on current data refer to _future_ responses
```

```
        legend(-6.6,3.6,bty="n", col=c(2,blues[4],blues[2]),cex=1.2, lty=c(1,1,1),  lwd=2,
               c("Fit","Estimation confidence interval at 95%","Prediction confidence interval at 95%"),)
```

## Multivariate linear model: mass loss as a function of predictors extracted from micrographs

A dataset composed of features extracted from micrographs (those more related with mass loss) in addition to the collagen mass loss is developed:

```
Dataset2$pesofresco2=c(COO$pesofresco2,CCT$pesofresco2,CO$pesofresco2)
Dataset2$Mode_log=log(Dataset2$Mode); 
Dataset2$Area_log=log(Dataset2$Area)
Dataset2$Circ._log=log(Dataset2$Circ.)
Dataset2$pesofresco2_log=log(Dataset2$pesofresco2)
rownames(Dataset2)=1:dim(Dataset2)[1]
```

A multivariate linear model is fitted and their parameters estimated to explain the collagen mass loss as a function of features extracted from micrographs (representative of cell growth and differentiation): intensity histogram mode, total area, and mean objects circularity, after logarithmic transformation, in order to linearize the effects of predictors on the response. In addtion, the effect of the treatment (addition of stem cells and osteogenic medium) is also studied (Grupo variable).

```
# Dataset3 is composed of those values where the logarithms of circularity, area and histogram mode are defined:
Dataset3=Dataset2[Dataset2$pesofresco2_log>0,]
rownames(Dataset3)=1:dim(Dataset3)[1]

# Multivariate regression model:
LinearModel.2 <- lm(pesofresco2_log ~ Grupo  + Mode_log+
                      Area_log + Circ._log,data=
                      Dataset3)
summary(LinearModel.2)
```

```
## 
## Call:
## lm(formula = pesofresco2_log ~ Grupo + Mode_log + Area_log + 
##     Circ._log, data = Dataset3)
## 
## Residuals:
##      Min       1Q   Median       3Q      Max 
## -0.51000 -0.10771  0.00220  0.09073  0.47149 
## 
## Coefficients:
##              Estimate Std. Error t value Pr(>|t|)    
## (Intercept) -14.48879    4.51639  -3.208  0.00317 ** 
## GrupoCCT     -0.18884    0.10757  -1.756  0.08938 .  
## GrupoCO      -2.03323    0.34882  -5.829 2.25e-06 ***
## Mode_log      3.32259    0.84990   3.909  0.00049 ***
## Area_log      0.14466    0.06642   2.178  0.03740 *  
## Circ._log    -0.06540    0.05725  -1.142  0.26228    
## ---
## Signif. codes:  0 '***' 0.001 '**' 0.01 '*' 0.05 '.' 0.1 ' ' 1
## 
## Residual standard error: 0.2291 on 30 degrees of freedom
## Multiple R-squared:  0.7673, Adjusted R-squared:  0.7285 
## F-statistic: 19.78 on 5 and 30 DF,  p-value: 1.082e-08
```

```
# The observations corresponding to studentized residuals greater than 2 or less than -2 are discarded due to they are susceptible to be outliers.

car::outlierTest(LinearModel.3,cutoff=Inf, n.max=Inf)
```

```
## No Studentized residuals with Bonferonni p < Inf
## Largest |rstudent|:
## [1] rstudent           unadjusted p-value Bonferonni p      
## <0 rows> (or 0-length row.names)
```

```
LinearModel.3 <- lm(pesofresco2_log ~ Grupo+ Circ._log 
                    + Mode_log+
                      Area_log 
                    ,data=Dataset3[-c(25,26,29,32,35),])
summary(LinearModel.3)
```

```
## 
## Call:
## lm(formula = pesofresco2_log ~ Grupo + Circ._log + Mode_log + 
##     Area_log, data = Dataset3[-c(25, 26, 29, 32, 35), ])
## 
## Residuals:
##       Min        1Q    Median        3Q       Max 
## -0.210003 -0.105933 -0.009659  0.066066  0.259035 
## 
## Coefficients:
##              Estimate Std. Error t value Pr(>|t|)    
## (Intercept) -17.03961    2.79442  -6.098 2.26e-06 ***
## GrupoCCT     -0.21504    0.06624  -3.247 0.003314 ** 
## GrupoCO      -2.17513    0.22599  -9.625 6.90e-10 ***
## Circ._log    -0.05988    0.03592  -1.667 0.107983    
## Mode_log      3.78039    0.52495   7.201 1.51e-07 ***
## Area_log      0.17599    0.04264   4.127 0.000357 ***
## ---
## Signif. codes:  0 '***' 0.001 '**' 0.01 '*' 0.05 '.' 0.1 ' ' 1
## 
## Residual standard error: 0.1398 on 25 degrees of freedom
## Multiple R-squared:  0.8872, Adjusted R-squared:  0.8647 
## F-statistic: 39.34 on 5 and 25 DF,  p-value: 4.607e-11
```

```
# Bootstrap 95% CI for regression coefficients 
library(boot)
```

```
## 
## Attaching package: 'boot'
```

```
## The following object is masked from 'package:car':
## 
##     logit
```

```
# function to obtain regression weights 
bs <- function(formula, data, indices) {
  d <- data[indices,] # allows boot to select sample 
  fit <- lm(formula, data=d)
  return(coef(fit)) 
} 
# bootstrapping with 1000 replications 
set.seed(4)
results <- boot(data=Dataset3[-c(25,26,29,32,35),], statistic=bs, 
                R=1000, formula=pesofresco2_log ~ Grupo + Circ._log  + Mode_log+
                  Area_log)

# get 95% confidence intervals 
boot.ci(results, type="bca", index=1) # intercept
```

```
## BOOTSTRAP CONFIDENCE INTERVAL CALCULATIONS
## Based on 1000 bootstrap replicates
## 
## CALL : 
## boot.ci(boot.out = results, type = "bca", index = 1)
## 
## Intervals : 
## Level       BCa          
## 95%   (-25.88, -10.40 )  
## Calculations and Intervals on Original Scale
```

```
boot.ci(results, type="bca", index=2) # CCT
```

```
## BOOTSTRAP CONFIDENCE INTERVAL CALCULATIONS
## Based on 1000 bootstrap replicates
## 
## CALL : 
## boot.ci(boot.out = results, type = "bca", index = 2)
## 
## Intervals : 
## Level       BCa          
## 95%   (-0.3460, -0.0782 )  
## Calculations and Intervals on Original Scale
```

```
boot.ci(results, type="bca", index=3) # CCO
```

```
## BOOTSTRAP CONFIDENCE INTERVAL CALCULATIONS
## Based on 1000 bootstrap replicates
## 
## CALL : 
## boot.ci(boot.out = results, type = "bca", index = 3)
## 
## Intervals : 
## Level       BCa          
## 95%   (-2.587, -1.641 )  
## Calculations and Intervals on Original Scale
```

```
boot.ci(results, type="bca", index=4) # ln(Circularity)
```

```
## BOOTSTRAP CONFIDENCE INTERVAL CALCULATIONS
## Based on 1000 bootstrap replicates
## 
## CALL : 
## boot.ci(boot.out = results, type = "bca", index = 4)
## 
## Intervals : 
## Level       BCa          
## 95%   (-0.1727,  0.0082 )  
## Calculations and Intervals on Original Scale
```

```
boot.ci(results, type="bca", index=5) # ln(Mode)
```

```
## BOOTSTRAP CONFIDENCE INTERVAL CALCULATIONS
## Based on 1000 bootstrap replicates
## 
## CALL : 
## boot.ci(boot.out = results, type = "bca", index = 5)
## 
## Intervals : 
## Level       BCa          
## 95%   ( 2.509,  5.387 )  
## Calculations and Intervals on Original Scale
```

```
boot.ci(results, type="bca", index=6) # ln(Area)
```

```
## BOOTSTRAP CONFIDENCE INTERVAL CALCULATIONS
## Based on 1000 bootstrap replicates
## 
## CALL : 
## boot.ci(boot.out = results, type = "bca", index = 6)
## 
## Intervals : 
## Level       BCa          
## 95%   ( 0.0652,  0.2489 )  
## Calculations and Intervals on Original Scale
```

```
# In order to measure the relative importance of each pedictor in the response, in terms of R^2, the relaimpo package is used as follows:

windows(15,7)
library(relaimpo)
boot <- boot.relimp(LinearModel.3, b = 100, type = c("lmg"), rank = TRUE,
                    diff = TRUE, rela = TRUE)
booteval.relimp(boot) # print result
```

```
## Response variable: pesofresco2_log 
## Total response variance: 0.1444701 
## Analysis based on 31 observations 
## 
## 5 Regressors: 
## Some regressors combined in groups: 
##         Group  Grupo : GrupoCCT GrupoCO 
## 
##  Relative importance of 4 (groups of) regressors assessed: 
##  Grupo Circ._log Mode_log Area_log 
##  
## Proportion of variance explained by model: 88.72%
## Metrics are normalized to sum to 100% (rela=TRUE). 
## 
## Relative importance metrics: 
## 
##                  lmg
## Grupo     0.55969629
## Circ._log 0.07561308
## Mode_log  0.13674673
## Area_log  0.22794390
## 
## Average coefficients for different model sizes: 
## 
##                1group     2groups     3groups     4groups
## GrupoCCT  -0.01317449 -0.09796714 -0.16703113 -0.21504292
## GrupoCO   -0.63589636 -1.15852814 -1.67916870 -2.17512543
## Circ._log  0.05364440 -0.09958465 -0.15045994 -0.05988144
## Mode_log  -1.08473092  0.91686149  2.68418545  3.78039335
## Area_log  -0.08337702 -0.03390108  0.04180355  0.17598807
## 
##  
##  Confidence interval information ( 100 bootstrap replicates, bty= perc ): 
## Relative Contributions with confidence intervals: 
##  
##                               Lower  Upper
##               percentage 0.95 0.95   0.95  
## Grupo.lmg     0.5597     A___ 0.3970 0.6167
## Circ._log.lmg 0.0756     __CD 0.0516 0.1993
## Mode_log.lmg  0.1367     _BCD 0.0939 0.3253
## Area_log.lmg  0.2279     _BC_ 0.1326 0.3138
## 
## Letters indicate the ranks covered by bootstrap CIs. 
## (Rank bootstrap confidence intervals always obtained by percentile method) 
## CAUTION: Bootstrap confidence intervals can be somewhat liberal. 
## 
##  
##  Differences between Relative Contributions: 
##  
##                                        Lower   Upper
##                        difference 0.95 0.95    0.95   
## Grupo-Circ._log.lmg     0.4841     *    0.2470  0.5575
## Grupo-Mode_log.lmg      0.4229     *    0.0693  0.5177
## Grupo-Area_log.lmg      0.3318     *    0.1333  0.4449
## Circ._log-Mode_log.lmg -0.0611         -0.2314  0.0885
## Circ._log-Area_log.lmg -0.1523         -0.2178  0.0202
## Mode_log-Area_log.lmg  -0.0912         -0.1852  0.1366
## 
## * indicates that CI for difference does not include 0. 
## CAUTION: Bootstrap confidence intervals can be somewhat liberal.
```

```
# Las dos variables son igual de importantes:
plot(booteval.relimp(boot))
```

```
# The DAAG package is used to evaluate the accuracy of the model predictions. A three fold crossvalidation process is implemented. The data is divided in three groups. In each interaction, two groups act as trainning sample and the remaining as test sample: the model is fitted with the training sample and the accuracy of predictions is evaluated by estimating the response value correspondeing to each observation of the test sample. This is repeated until all the groups act as test sample.

library(DAAG)
a=cv.lm(data=Dataset3, LinearModel.3, m=3)
```

```
## Analysis of Variance Table
## 
## Response: pesofresco2_log
##           Df Sum Sq Mean Sq F value Pr(>F)    
## Grupo      2   3.79   1.894   36.09  1e-08 ***
## Circ._log  1   0.53   0.526   10.03 0.0035 ** 
## Mode_log   1   0.63   0.628   11.97 0.0016 ** 
## Area_log   1   0.25   0.249    4.74 0.0374 *  
## Residuals 30   1.57   0.052                   
## ---
## Signif. codes:  0 '***' 0.001 '**' 0.01 '*' 0.05 '.' 0.1 ' ' 1
```

```
## 
## fold 1 
## Observations in test set: 12 
##                      2      3      6     14   16    18       21     22
## Predicted        3.636  3.921  4.052  3.875 3.85  4.15  4.10628  4.366
## cvpred           3.674  4.145  4.093  3.931 3.86  4.18  4.20470  4.432
## pesofresco2_log  3.575  3.953  3.945  3.650 3.99  4.04  4.19585  4.256
## CV residual     -0.099 -0.192 -0.149 -0.281 0.13 -0.14 -0.00884 -0.176
##                    25    29    34     35
## Predicted       3.308 3.246 3.493  3.381
## cvpred          3.194 3.169 3.454  3.317
## pesofresco2_log 3.718 3.718 3.718  2.871
## CV residual     0.524 0.549 0.264 -0.447
## 
## Sum of squares = 1.06    Mean square = 0.09    n = 12 
## 
## fold 2 
## Observations in test set: 12 
##                      1      5     7     9      10     12     15     19
## Predicted        3.712  4.118 4.041 4.112  4.3186 4.2374 3.9904 4.0847
## cvpred           3.733  4.118 4.031 4.121  4.3475 4.2362 3.9573 4.0600
## pesofresco2_log  3.575  3.987 4.136 4.340  4.2526 4.2651 3.9909 4.1197
## CV residual     -0.157 -0.131 0.105 0.219 -0.0949 0.0289 0.0335 0.0597
##                    24     26      31     32
## Predicted       4.033  3.336  3.1698  3.558
## cvpred          4.005  3.444  3.2568  3.652
## pesofresco2_log 4.292  2.871  3.1583  3.158
## CV residual     0.287 -0.574 -0.0986 -0.493
## 
## Sum of squares = 0.78    Mean square = 0.07    n = 12 
## 
## fold 3 
## Observations in test set: 12 
##                      4      8    11    13      17      20    23      27
## Predicted       3.9119 4.2002 4.265  3.35  4.1718  4.2883 4.101  3.2668
## cvpred          3.9314 4.1643 4.225  3.66  4.1331  4.2588 4.032  3.1937
## pesofresco2_log 3.9533 4.2041 4.340  3.25  4.0684  4.2225 4.292  3.1583
## CV residual     0.0219 0.0398 0.115 -0.41 -0.0647 -0.0363 0.259 -0.0355
##                      28    30      33    36
## Predicted        3.2407 3.388 3.32016 3.483
## cvpred           3.2308 3.307 3.37487 3.403
## pesofresco2_log  3.1583 3.718 3.38139 3.564
## CV residual     -0.0725 0.411 0.00653 0.161
## 
## Sum of squares = 0.46    Mean square = 0.04    n = 12 
## 
## Overall (Sum over all 12 folds) 
##     ms 
## 0.0638
```
